# Global status of research on fertility preservation in male patients with cancer: A bibliometric and visual analysis

**DOI:** 10.1016/j.heliyon.2024.e33621

**Published:** 2024-06-25

**Authors:** Chuan Huang, Xi-Ren Ji, Zeng-Hui Huang, Rui-Jun Wang, Li-Qing Fan, Wen-Bing Zhu, Qiang Luo, Qing-Li

**Affiliations:** a The Institute of Reproductive and Stem Cell Engineering, Basic Medicine College, Central South University, Changsha, Hunan, China; b Human Sperm Bank, The Reproductive & Genetic Hospital of CITIC-XIANGYA, Changsha, China; c Department of Oncology, The Second Affiliated Hospital of Nanchang University, Nanchang, China

**Keywords:** Cancer, Male fertility preservation, Bibliometrics, CiteSpace, WoSCC

## Abstract

**Background:**

Recently, male fertility preservation before cancer treatment has become more prevalent. The research in this field has progressed over time, with some studies having a major impact and providing guidance for further research. However, the trends and hotspots of research on fertility preservation in male cancer patients may have changed; exploring them is essential for relevant research progress.

**Design:**

We extracted relevant studies from the Web of Science Core Collection database, capturing information on the countries of study, affiliations, authors, keywords, as well as co-citations of references and journals. To identify publication trends, research strengths, key subjects, prominent topics, and emerging areas, we conducted a bibliometric analysis using CiteSpace.

**Results:**

We included 3201 articles on fertility preservation in male cancer patients published over January 1999 to December 2023 were included. Although the relevant research growth rate was slow initially, the number of publications increased annually. Of all study countries, the United States, Germany, and Japan reported the earliest studies; the United States published the highest number of relevant studies. The US institutions remained at the forefront for all 25 years, and the US researcher Ashok Agarwal published the most articles. Literature co-citation analyses indicated a transformation in the study participants; they comprised a younger demographic (i.e., a large number of adolescent male patients underwent fertility preservation); moreover, fertility preservation techniques evolved from sperm cryopreservation to testicular tissue cryopreservation. Research on reproductive outcomes of sperm cryopreservation was the recent hotspot in male fertility preservation research, and the impact of immunotherapy and checkpoint inhibitors on male fertility requires further research.

**Conclusions:**

Male fertility preservation will be a major future research focus, with closer connections and collaborations between countries and organizations. Our results present the historical data on the development of research on male fertility preservation in cancer patients, providing relevant insights for future research and development in this study area.

## 1 Introduction

Global cancer incidence is increasing, with 19.3 million new cases reported in 2020. Compared with women, men demonstrate a 19 % higher rate of all cancers combined; however, this number may vary significantly in different areas worldwide [1]. Over the past few years, breakthroughs in cancer treatment have resulted in higher survival rates for younger patients with cancer, with approximately 80 % of them now expected to be cured long-term [2]. However, cancer treatment methods such as surgery, radiotherapy, and chemotherapy may lead to temporary or permanent fertility reduction in both males and females.

Before treatment initiation, physicians should discuss potential fertility risks associated with chemotherapy, radiotherapy, or both and explore possible preservation options with cancer patients and their families. Sperm cryopreservation is a safe and established method for preserving fertility in male cancer patients prior to commencing gonadotropic treatments. Consequently, it is advisable for these patients and their caregivers to be regularly directed to a fertility specialist [3]. However, in prepubertal boys with no mature sperms, sperm cryopreservation is difficult [4]. Nevertheless, studies have reported various methods for fertility maintenance in prepubertal boys; however, they remain in the experimental stage [5]. Recently, male fertility preservation before cancer treatment has become more prevalent; Moreover, relevant studies have developed male fertility preservation methods for cancer patients and reported their characteristics [[6], [7], [8]]. The research in this field has progressed over time, with some studies having a major impact and providing guidance for further research. However, to the best of our knowledge, all literature reviews in this field primarily provide qualitative summaries and only provide sampling insights into certain research. Therefore, there is a need to conduct both quantitative and qualitative visual analyses of the literature concerning fertility preservation in male cancer patients from a macroscopic viewpoint.

To address this gap, we used bibliometrics to analyze the dynamics of literature production. Bibliometrics is a method that quantitatively measures published literature through bibliometric data analysis to gain insight into the development of a particular field of study [9]. Meanwhile, it can analyze large quantities of publications at both macroscopic and microscopic levels, while also being domain-independent [10]. CiteSpace is a powerful instrument that facilitates the observation and evaluation of the trends and designs in scholarly articles. It is particularly advantageous for understanding the network structures in research fields and tracing research hotspots [11]. Thus far, no study has analyzed the scientific patterns related to fertility preservation in male cancer patients. Hence, this study represents the inaugural utilization of CiteSpace for the qualitative examination of literature concerning fertility preservation in male cancer patients within the last 25 years, with a specific emphasis on evolutionary trends and prominent research areas.

## 2 Materials and methods

### 2.1 Data source and collection

We searched the Web of Science Core Collection (WoSCC) database for literature on fertility preservation in male cancer patients published from January 1, 1999, to December 31, 2023. Because WoSCC is a globally renowned database, the included literature has the details required for bibliometric software and guarantees research accuracy. We used the following main search terms: “freezing” or “banking” or “cryostorage” or “storage” or “cryopreservation” or “freeze” or “fertility preservation” or “fertility preservation” and“cancer” or “tumor” or “malignancy” or “neoplasm” and“male” or “man” or “men.” Only original research and review articles regarding fertility preservation in male cancer patients published in English with full text available were included. Nonetheless, guidelines, editorials, and statements were not considered. Refer to Table S1 for a summary of our search strategy.

J.X.R. and H.Z.H. conducted individual searches on the WoSCC database to identify pertinent literature. The retrieved works were downloaded and saved in the “full record with cited references” format, with duplicates removed. The analysis was based on a final sample comprising 3201 articles.

### 2.2 Data analyses and visualization

CiteSpace (version 6.3. R2), used in this study, is a JavaScript-based software program developed by Prof. Chaomei Chen at Drexel University [12]. The method is capable of identifying and analyzing trends and shifts within a scientific field over time. It is utilized to explore key literature indicators, such as countries, authors, institutions, keywords, and co-citations of references. This method illustrates the evolution of a research domain, the emergence of novel study areas, and forecasts future research trajectories. In this study, the selection criteria [13] involved selecting the top n per slice (i.e., the highest n data points from each slice were extracted to form the final network). Furthermore, the analysis also took into account various factors such as network characteristics (N = number of network nodes; E = number of links; Density = network density), Largest CC (representing the maximum co-citation or co-occurrence frequency), Pruning (referring to the network pruning technique), Modularity Q (indicating the clustering module value; Q > 0.3 signifies a significant clustering structure), and Weighted Mean Silhouette S (referring to the average silhouette value of the cluster; S > 0.5 and S > 0.7 denote reasonable and strong clustering, respectively). Microsoft Office Excel (version 2019) was employed to construct a visualization displaying the yearly distribution of publications and the growth trends of the journals.

## 3 Results

A total of 3201 studies on fertility preservation in male cancer patients, published between 1999 and 2023, were identified through our WoSCC database search (Fig. S1). These studies garnered a total citation count of 95,882, which, after excluding self-citations, amounts to 87,480. On average, each study was cited 29.95 times, with an Hirsch Index (H-index) of 126 for this period. Over the 25-year period, there was a predominantly upward trend in yield, with the highest yield observed in 2022 (n = 238) and the lowest yields in 2001 and 2002 (n = 65).

### 3.1 Country and region analysis

**Fig. 1:**
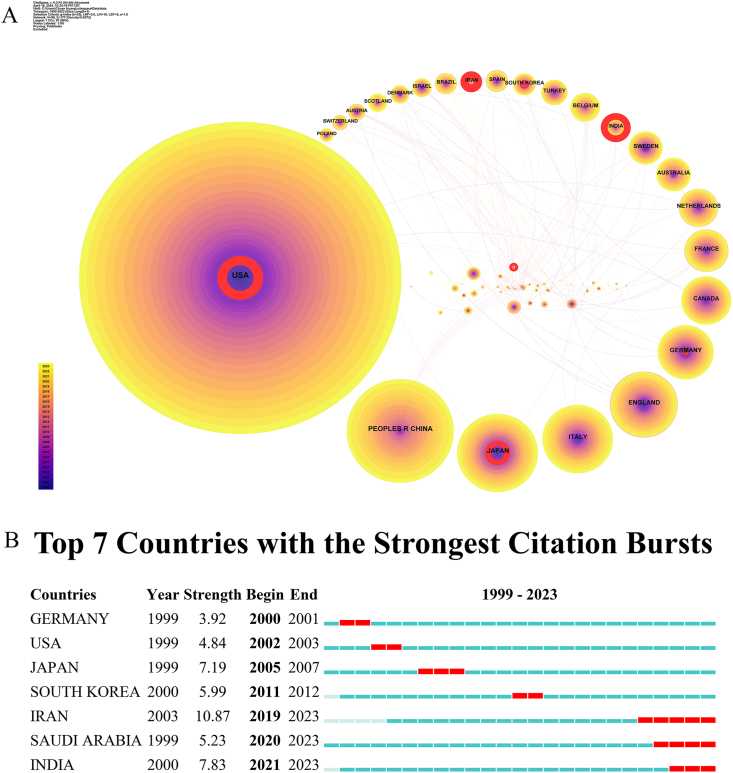
Analysis of countries engaged in research on fertility preservation in male cancer patients. (A) Network diagram showing country links [Timespan: 1999–2023; Slice Length: 1; Selection Criteria: top 50 per slice; Network: N = 98, E = 177; Density: 0.0372; Largest CC: 87 (88 %); Nodes Labeled: 1.0 %; Pruning: Pathfinder]. (B) Top 7 countries with burst period after 1999.

Between 1999 and 2023, publications concerning fertility preservation in male cancer patients were distributed across 98 countries or regions. (Fig. S2). Of these countries or regions (Fig. 1A), the United States demonstrated the highest volume of publications (n = 1015, 23.8 %), followed by China (n = 337, 7.9 %), Japan (n = 254, 6.0 %), Italy (n = 222, 5.2 %), England (n = 212, 5.0 %), Germany (n = 176, 4.1 %), Canada (n = 157, 3.7 %), France (n = 137, 3.2 %), the Netherlands (n = 122, 2.9 %), and Australia (n = 109, 2.6 %). Together, these countries accounted for 64.37 % of all publications (n = 4265). This implies that the United States is at the forefront of research in this field on a worldwide level. Our burst analysis (Fig. 1B) revealed that fertility preservation in male cancer patients was a hot topic in the United States between 2002 and 2003. From 2019 onward, Iran, Saudi Arabia, and India demonstrated a surge in research on this topic, which was maintained until now. Moreover, extensive collaboration was noted between different countries or regions. However, the United States and Japan, two of the top three, were only engaged in cooperation with a few regions. In particular, the United States collaborated with the Netherlands and Canada, while Japan collaborated with Scotland and Belgium.

### 3.2 Institutional analysis

**Fig. 2:**
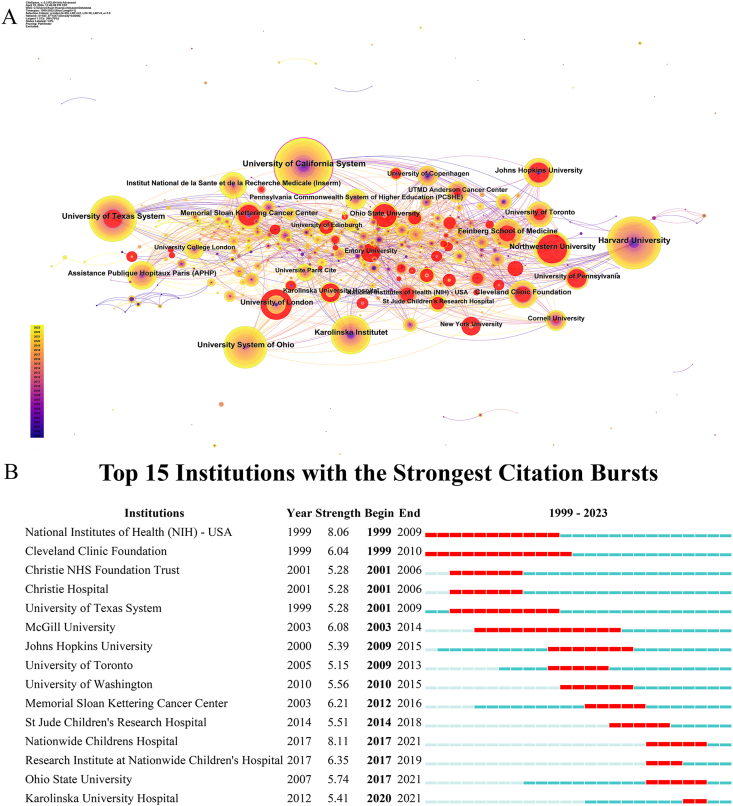
Analysis of institutions involved in research on fertility preservation in male cancer patients. (A) Network diagram showing institution links [Timespan: 1999–2023; Slice Length: 1; Selection Criteria: Top 50 per slice; Network: N = 547, E = 1417; Density: 0.0095; Largest CC: 386 (70 %); Nodes Labeled: 1.0 %; Pruning: Pathfinder]. (B) Top 15 institutions with burst period after 1999.

This study included studies from 547 institutions. Among these institutions, the top 10 in publication volume included nine universities and one hospital, with the University of California System leading with 86 publications. Harvard University (n = 80), University of Texas System (n = 70), University System of Ohio (n = 64), Karolinska Institute (n = 59), Northwestern University (n = 54), University of London (n = 46), Feinberg School of Medicine (n = 45), Johns Hopkins University (n = 44), and Assistance Publique Hopitaux Paris (APHP) (n = 43). seven of these institutions are located in the United States and have strong connections; Despite the University of California System having the highest number of publications (2.4 %) and possessing substantial expertise in fertility preservation for male cancer patients, the average publication year for this institution falls in an earlier period. Based on the data presented in Fig. 2A, it is evident that inter-institutional collaboration surpassed international collaboration. Burst analysis revealed that esteemed institutions were actively involved in studies related to fertility preservation in male cancer patients. Additionally, the influence of institutions such as Nationwide Childrens Hospital, Ohio State University, and Karolinska University Hospital has increased recently (Fig. 2B).

### 3.3 Author and co-cited author analysis

**Fig. 3:**
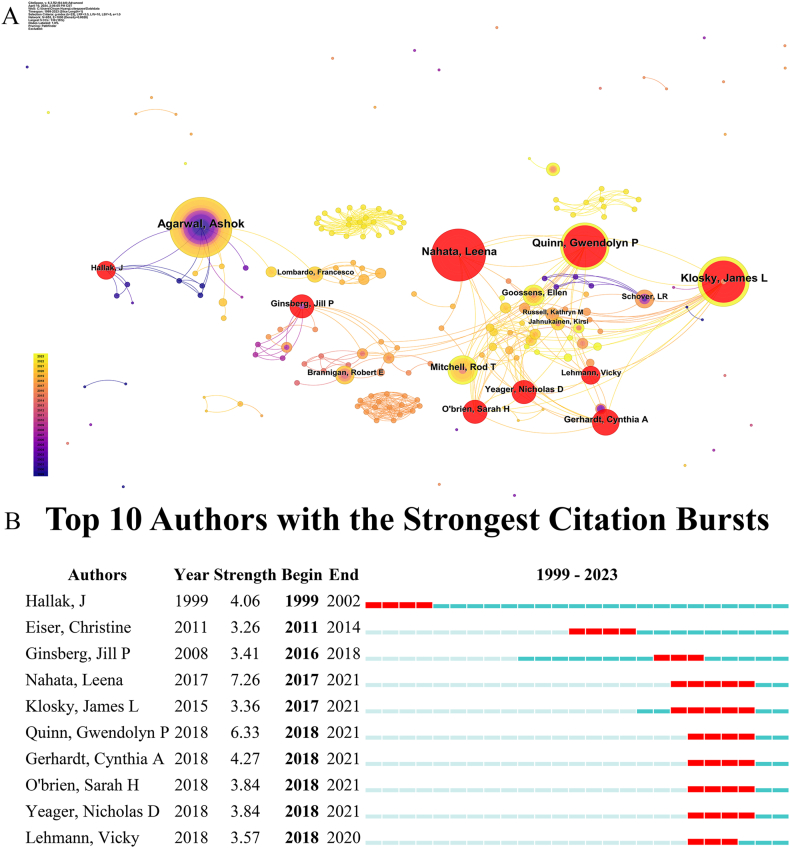
Analysis of authors involved in research on fertility preservation in male cancer patients. (A) Network diagram showing author links [Timespan: 1999–2023; Slice Length: 1; Selection Criteria: Top 50 per slice; Network: N = 853, E = 1000; Density: 0.0028; Largest CC: 139 (16 %); Nodes Labeled: 1.0 %; Pruning: Pathfinder]. (B) Top 9 authors with burst period after 1999.

Based on Lotka's Law, it is expected that among 853 authors, around 8 authors would have published more than 10 papers. Nevertheless, the actual number of authors meeting this criterion is only 6. Table S2 lists the 6 most productive authors in literature related to fertility preservation in male cancer patients; Ashok Agarwal (n = 23) was the most productive author, followed by Leena Nahata (n = 20), Klosky, James L (n = 19), and Gwendolyn P Quinn (n = 18). Among the top 6 co-cited authors, Leslie R Schover (n = 270) ranked first, followed by Lee Stephanie J (n = 233), Oktay Kutluk (n = 213), and Loren Alison W (n = 206). In additional, Agarwal, Ashok (n = 195) was the six of top 10 co-cited authors. It's worth noting that Agarwal, Ashok's H-index of 101 signifies that 101 of his papers have each garnered at least 101 citations, highlighting his notable influence in the field relative to other authors. In Fig. 3A, the authors' relationships are depicted, with the size of each node representing the number of published articles by the author, and the width of the connecting lines indicating the level of collaboration between them. The author network resembled a constellation due to the strong ties between countries and institutions, with only a few scholars showing significant collaborative partnerships, primarily at an institutional level. According to our burst analysis (Fig. 3B), Leena Nahata, James L Klosky and Gwendolyn P Quinn's works were hot topics between recent years.

### 3.4 Keyword analysis

**Fig. 4:**
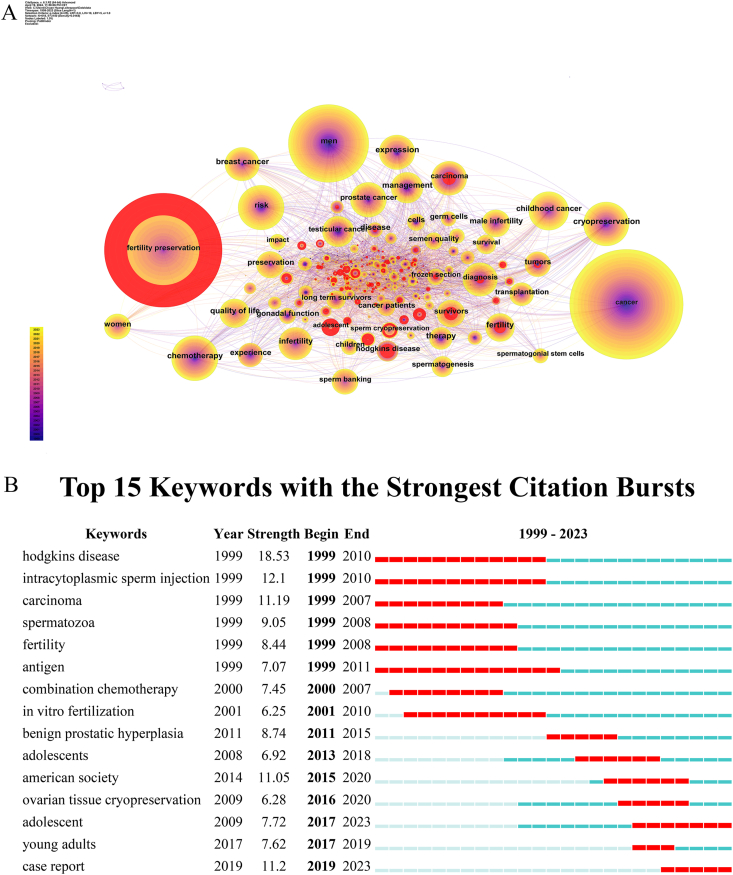
Analysis of keywords involved in research on fertility preservation in male cancer patients. (A) Network diagram showing keywords links [Timespan: 1999–2023; Slice Length: 1; Selection Criteria: Top 50 per slice; Network: N = 618, E = 3150; Density: 0.0165; Nodes Labeled: 1.0 %; Pruning: Pathfinder]. (B) Top 15 keywords with burst period after 1999.

In a paper, keywords paper provides an accurate representation of the research focus of the field, thus reflecting the main theme of the paper. Moreover, burst keywords can provide valuable insight into emerging and innovative study topics. Here, CiteSpace extracted 618 keywords, and 25 of them occurred more than 100 times. These include “fertility preservation,” “cancer,” “men,” “chemotherapy,” “risk,” “cryopreservation,” “childhood cancer,” “prostate cancer,” “expression,” “carcinoma” and so on (Fig. 4A). The term “fertility preservation” is the most frequently utilized word, occurring 491 times. Following Zipf's Law, the expected frequency for the second-ranked term, “cancer,” would be approximately 245.5 occurrences, yet it is observed 473 times. Likewise, for the third-ranked term, “men,” the projected frequency is around 163.67 occurrences, whereas it is found 335 times in reality. However, this keyword analysis has limitations: use of synonymous keywords can lead to a decrease in the frequency of a particular keyword. Fig. 4B illustrates the transformation of hotspots in research on male fertility preservation over the past 25 years, as demonstrated by the change in keyword bursts. Of the top 15 keywords with bursts, “Hodgkin's disease” demonstrated the highest burst strength (18.53) and longest burst duration (12 years). In other words, the study on fertility preservation in male cancer patients is significantly impacted by Hodgkin's disease, owing to its prevalence as the most common ailment among male cancer patients requiring fertility preservation. The keywords “young adult” and “adolescent” received more attention at the beginning of 2017 and 2020, respectively; this popularity was retained until 2023. These results indicated that male cancer patients who underwent fertility preservation were typically younger.

### 3.5 Co-cited reference and time zone analysis

**Fig. 5:**
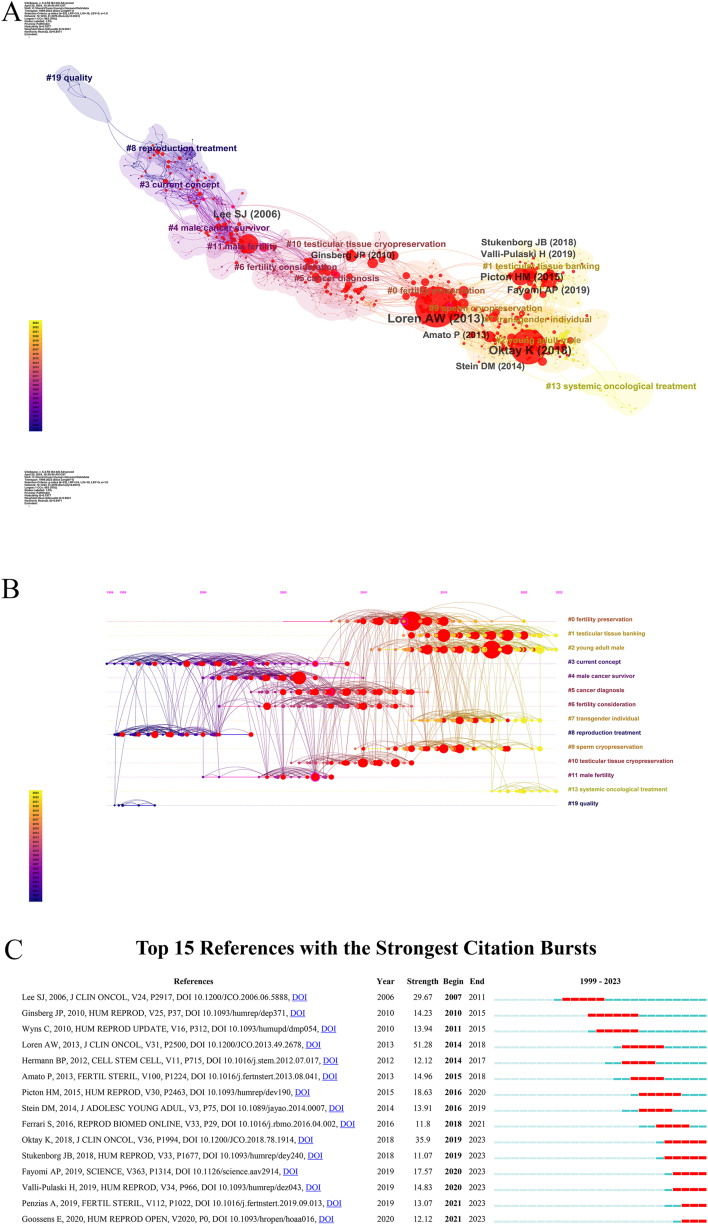
Analysis of the cited articles. (A) Knowledge map of the cited literature [Timespan: 1999–2023; Slice Length: 1; Selection Criteria: Top 50 per slice; Network: N = 1223, E = 3076; Density: 0.0041; Largest CC: 962 (78 %); Nodes Labeled: 1.0 %; Pruning: Pathfinder; Modularity Q: 0.7977; Weighted Mean Silhouette S: 0.9031]. (B) Time axis map of the cited literature [Timespan: 1999–2023; Slice Length: 1; Selection Criteria: Top 50 per slice; Network: N = 1223, E = 3076; Density: 0.0041; Largest CC: 962 (78 %); Nodes Labeled: 1.0 %; Pruning: Pathfinder; Modularity Q: 0.7977; Weighted Mean Silhouette S: 0.9031]. (C) Top 15 References in publishing research with burst period after 1999.

Co-cited references not only illuminate the foundational literature that has had a substantial impact on shaping a field but also indicate the changing research emphasis. Fig. 5A underscores the prominent authors and years in terms of citation frequency. Here, clustering was based on the degree of association among articles, divided into 19 categories. The lighter yellow shade indicates a more recent time frame, with the average year representing the median publication year of the cited literature. The silhouette coefficient, denoted as “S,” quantifies the degree of attention focused on a specific topic. A value of S > 0.7 signifies strong clustering. The silhouette coefficients of all clusters exceeded 0.85, as detailed in Table S3. The category with the highest number of published articles was #0, with the most prevalent keyword being “fertility preservation.” The earliest research areas in male cancer patients' fertility preservation were concentrated in research cluster #19 (quality), focusing primarily on quality of life and adult male cancer patients. Later, clusters developed into #8 (reproduction treatment), #3(current concept), #4(male cancer survivor), #5(cancer diagnosis), #6 (fertility consideration) and #11 (male fertility), began to be studied in the impact of cancer treatment on fertility, seeking a balance between improving male cancer survival rates and preserving male fertility. However, the studies primarily centered on adult male cancer patients. Subsequently, male fertility preservation methods such as testicular tissue cryopreservation and sperm cryopreservation were introduced, expanding the participant to include young adult male patients. Recently, cluster #13, which focuses on systemic oncological treatment, has emerged as a relatively independent cluster, which may be related to the increasingly diverse cancer treatment options.

A timeline map was created by developing a co-citation network graph using temporal nodes (refer to Fig. 5B). The size of the circles on the map corresponds to the number of citations, with larger circles indicating a higher number of citations received by the articles. The timeline map reveals peaks in research activity on fertility preservation during the periods of 2008–2020 and 2010–2023. In 2013 and 2018, two influential clinical guidelines for cancer patients were published, with the former article holding the distinction of being the most cited in this field. It is noteworthy that the top 10 most-cited articles in this field all trace their origins back to these two time periods.

Fig. 5C displays the top 15 references exhibiting the most pronounced citation bursts. the first two of them happened in 2006: “American Society of Clinical Oncology recommendations on fertility preservation in cancer patients” [14]. The paper “Fertility preservation for patients with cancer: American Society of Clinical Oncology clinical practice guideline update” [15], published by Loren et al. in the *Journal of Clinical Oncology* in 2015, demonstrated the strongest burst (strength = 51.28), which lasted until 2018. The paper “Fertility Preservation in Patients with Cancer: ASCO Clinical Practice Guideline Update” [3], published by Oktay et al. in the *Journal of Clinical Oncology* in 2018, also demonstrated a strong burst (strength = 35.9), which lasted until 2018.

### 3.6 Research outputs between different journals

**Fig. 6:**
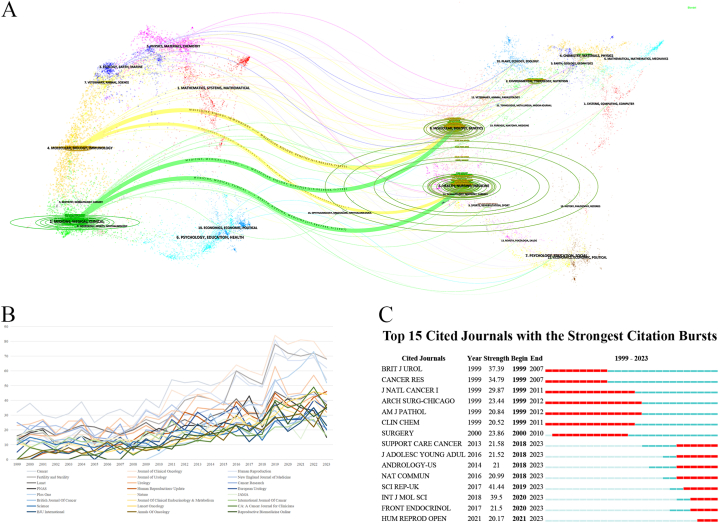
(A) Dual-map overlay of journals. The citing papers are listed on the left, whereas the cited papers are presented on the right; the curve between them presents the citation relationship. Different colors denote journals focused on different subjects. The lengths of the ellipse's vertical and horizontal axis are proportional to the counts of papers published in the journals and by the authors, respectively. (B) Annual growth trends of the journals. (C) Top 15 cited journals with burst period after 1999. (For interpretation of the references to color in this figure legend, the reader is referred to the Web version of this article.)

The geographical region depicted on the left in Fig. 6A is related to the journal that made the citation, whereas the map on the right corresponds to the cited journal; the various disciplines are differentiated on the basis of the respective line colors. In our observation, journals referencing studies on fertility preservation in male cancer patients predominantly emphasized molecular biology, immunology, and clinical medicine. Conversely, the sources cited primarily originated from journals in the fields of molecular biology, genetics, nursing, and medicine. Consequently, the need for interdisciplinary management in fertility preservation in male cancer patients was apparent because it was noted to be closely related to both basic and clinical subjects, requiring improvements in the future. According to the data in Fig. 6B and Table S4, Applying Bradford's Law principles to analyzing the smallest number of journals that concentrate one-third of the publications. Hence, the 24 core journals that contribute to a significant portion of publications in the field. The preeminent publication in the field was *Cancer*, with the most rapid publication rate, followed by journals such as the *Journal of Clinical Oncology* and *Journal of Urology, Human Reproduction, Fertility and Sterility* and so on. The articles published in these journals are presumed to have superior academic caliber. According to the burst analysis (Fig. 6C), the Support *Care Cancer*, *Andrology*, *Nature Communications*, *Journal of Adolescent and Young Adult Oncology*, *Scientific Reports*, *Human Reproduction Open*, *Frontiers in Endocrinology*, and *International Journal of Molecular Sciences* were hot cited journals.

## 4 Discussion

Here, we, for the first time, performed a scientometric analysis focused on fertility preservation in male cancer patients. In particular, we used CiteSpace to analyze 3201 English-language papers from 98 countries published between 1999 and 2023. Our bibliometric analysis offers a comprehensive examination of the output dynamics, research trends, key topics, hotspots, and frontiers within the realm of fertility preservation for male cancer patients. Our results may aid scholars in gaining a quick overview of the related research, formulating their research ideas, and staying informed of the current research status.

Research on fertility preservation in male cancer patients over the previous 25 years has been conducted from various perspectives. Despite the slow growth rate, the number of articles on male fertility preservation increased annually. T The findings suggest a growing focus on fertility preservation research in male cancer patients, with Germany, the United States, and Japan pioneering early studies in this field. The United States stands out for having the highest publication output. The burst analysis of study institutions demonstrated that for 25 years, the United States was at the forefront of the development of fertility preservation in male cancer patients. Nevertheless, in recent years, Karolinska University Hospital and Karolinska Institute, both in Sweden, have demonstrated considerable progress. Although they began relevant research relatively later, rapid advancements were made at the Swedish institutions.

Upon analyzing authors and their co-citations, it became clear that Ashok Agarwal, based in the United States, had the highest number of published articles, underscoring the breadth of his research in the field. In addition, numerous scientometric studies have identified Ashok Agarwal as the key contributor to male infertility research. He covered topics such as sperm DNA fragmentation [16], varicocele [17], and oxidative stress [18] in male infertility, as well as the effects of antioxidants [19], androgen replacement therapy [20], and innovative tools such as proteomics [21] for male infertility investigation and management. Furthermore, Agarwal, Ashok's H-index was 101, highlighting his notable influence in the lots of fields relative to other authors. Leslie R. Schover, a distinguished American author, ranks first in co-citations, highlighting her significant influence and academic standing in the field of male fertility preservation. In the *Journal of Clinical Oncology*, Schover authored two articles examining the knowledge, attitudes, and experiences of oncologists and male cancer patients concerning cancer-related infertility and sperm banking. The author, consequently, suggested that sperm banking should be an option for all men at risk of infertility due to cancer treatment to allow them to make an informed decision. To ensure that oncologists do not use biased criteria when selecting patients, having clear practice standards in place is crucial [[22], [23]].

Our burst analysis revealed that the topics of discussion concerning fertility preservation in male cancer patients significantly shifted in recent years, particularly regarding the concerns and objectives addressed and methods used. In the past, the field of oncology was mainly associated with the current concepts of cancer treatment and survival without considering many other factors. However, cancer treatment often results in reproductive impairment or infertility in the surviving male patients. This can cause substantial emotional distress, including stress, anxiety, and depression, as well as worsen the quality of life [24,25]. Over the last twenty years, advancements in cancer diagnostics and treatments have significantly improved survival rates among men of reproductive age. The changing landscape of fertility and sexual function has raised important considerations for healthcare providers and cancer patients at the beginning of treatment. Data from the National Center for Health Statistics shows an increase in the 5-year relative survival rate for all cancers, climbing from 49 % for patients diagnosed in the mid-1970s to 68 % for those diagnosed between 2012 and 2018. This improvement resulted in a 33 % reduction in overall mortality by 2020 [26]. The guidelines issued by the American Society for Clinical Oncology in 2006 and 2013 advised healthcare providers to be prepared to address fertility preservation choices, make referrals to reproductive specialists, or both for potential cancer patients [14,15]. The latest revisions to these guidelines, released in 2018, provide up-to-date recommendations on fertility preservation options for cancer patients preparing for treatment [3]. Moreover, these guidelines ranked among the top 15 references with the most significant citation bursts, underscoring the critical importance of offering healthcare providers guidance on fertility preservation for male cancer patients.

Our literature clustering analysis indicated a change in the study participants, with the number of participants being younger and more adolescent. Currently, there is inadequate research on fertility preservation in male patients, leading to a significant number of adolescent cancer survivors experiencing profound regret for not being able to cryopreserve their sperm before undergoing cancer treatment [27]. Sperm cryopreservation provides adult men who may become infertile with an opportunity to pursue parenthood. However, this approach is not applicable to prepubertal boys. Hence, literature clustering analysis also revealed that popular fertility preservation methods assessed in relevant studies have changed from sperm cryopreservation to testicular and spermatogonial stem cell cryopreservation. Nevertheless, in clinical practice, sperm cryopreservation remains the most commonly used, well-established technique for male fertility preservation worldwide. Compared with spermatogonial stem cell cryopreservation, testicular tissue cryopreservation is the preferred choice. This is because, in testicular tissue cryopreservation, spermatogonial stem cells are cryopreserved along with the surrounding supportive environment, which allows for either transplantation of the spermatogonial stem cells or the whole tissue, enabling in vitro spermatogenesis after thawing [28]. A recent survey of 15 facilities providing testicular tissue cryopreservation for prepubertal males revealed that dimethyl sulfoxide was the favored cryoprotectant in 11 of these centers, with controlled slow freezing being the preferred method of freezing [29]. Although testicular tissue grafting and xenografting technology has been successfully reproduced in various mammalian species, achieving full spermatogenesis from transplanted human tissues remains elusive. Furthermore, no research documents the transplantation of cryopreserved human testicular tissue resulting in sperm and offspring generation [30,31]. Further research is necessary to enhance testicular cryopreservation techniques for clinical applications. Additionally, a significant constraint of testicular tissue cryopreservation and transplantation in cancer patients is the potential risk of reintroducing malignant tumor cells to the transplanted tissue [32]. This necessitates comprehension of the extent, safety, and practicality of testicular tissue grafting in cancer patients.

In the current landscape of oncology, treatment methods have advanced significantly beyond traditional radiation and chemotherapy to include immunotherapy and checkpoint inhibitors, therapy. This shift has brought systemic oncological treatment to the forefront of attention and concern. Despite progress in cancer treatment [33], the impact of immunotherapy and checkpoint therapy on male fertility is not well understood. Some data suggest that checkpoint inhibitors may induce primary hypogonadism, albeit based on limited and anecdotal evidence from case reports or series. Notably, two case reports documented incidences of orchitis and epididymal-orchitis in individuals undergoing treatment with anti-PD1/anti-CTLA4 and anti-PD1 agents, respectively [34]. Underscoring the importance of focused research in this field, it is critical for the scientific community to escalate inquiries into the effects of these innovative therapies on male reproductive health. This is vital for developing effective strategies for preserving fertility during cancer treatment.

In recent years, the keywords “intracytoplasmic sperm injection,” “in vitro fertilization,” “case report,” “transplantation,” “diagnosis,” and “management” have emerged as significant in the field of male fertility preservation. This suggests that sperm cryopreservation's impact on reproductive outcomes is a current focal point.” Sperm cryopreservation is deemed effective for preserving fertility in male cancer patients, as indicated by several reviews [[35], [36]]. Assisted reproductive technology is crucial for fertility preservation and successful pregnancy in this population, with ICSI yielding superior clinical results compared to IVF and IUI [37]. Notably, the major keywords also included “Hodgkin's disease,” “testicular cancer,” “prostate cancer,” and “childhood cancer,” indicating that the primary cancer types observed in male patients seeking fertility preservation are tumors of the urinary and blood systems. These tumors demonstrate an early onset age but with a favorable prognosis [38,39], and these tumors patients are concerned about their future fertility capacity.

Defective spermatogenesis, a common consequence of Hodgkin's lymphoma[40] and testicular malignancies [41], leads to a decline in sperm quality. The World [42] guidelines report that approximately 48.0 % and 23 % of patients with testicular and hematological malignancies, respectively, present with oligozoospermia [42]. Thus, the preservation of fertility in male cancer patients necessitates the collaborative engagement of oncologists, reproductive medicine specialists, and embryologists.

Within the last 25 years, this study stands as the first bibliometric analysis to scrutinize research on male fertility preservation. However, this study has some limitations. First, we only used the WOSCC database to search for relevant articles; However, we did not incorporate data from additional sources like PubMed, Cochrane Library, and Google Scholar. While the WOSCC is extensive and dependable, there is a possibility of certain articles being missed. Second, we exclusively searched for literature published in English, which potentially led to skewed results. Third, recent high-quality articles may not have focused on male fertility preservation, as indicated by the low citation rates, highlighting the need for future research updates. Then, the strategies for literature search on male fertility preservation in cancer patients may introduce an initial search bias, which mainly affects the interpretation of relevant topics and thematic trends of the subject under study. Finally, the retrospective nature is an inherent limitation of the bibliometric method used here. Nevertheless, our analysis encompassed most articles on male fertility preservation, affording insight into current research trends and hotspots.

## 5 Conclusions

In this study, we performed a thorough examination of publication patterns related to fertility preservation in male cancer patients, revealing a consistent rise over the 25-year observation period. Our findings underscore the critical importance of offering pertinent guidance to healthcare professionals in the realm of fertility preservation for male cancer patients. In addition, male fertility preservation may be a major research focus in the future, with closer ties and collaborations between countries and organizations. In the future, research participants are expected to become more diverse, mainly including younger individuals such as adolescent male patients. Clinical research will continue to prioritize studies on the reproductive outcomes of sperm cryopreservation in cancer patients. The number of such studies is expected to rise, focusing on newer cryopreservation methods like testicular tissue and spermatogonial stem cell cryopreservation. The impact of immunotherapy and checkpoint therapy on male fertility remains inadequately understood, underscoring the necessity for targeted research in this domain.

In general, we identified the key countries, institutions, authors, keywords, and citation relationships related to research on fertility preservation in male cancer patients. This data may be used to rapidly trace the historical advancements related to male fertility preservation, gain insights into the upcoming progress, and direct future research practices. Our findings are expected to be a source of motivation for further male fertility preservation research.

## Ethical approval

Not applicable.

## Funding

This work was supported by grants from the 10.13039/501100012166National Key R&D Program of China (2022YFC2702700), 10.13039/501100004735Natural Science Foundation of Hunan Province (2024JJ6725, 2022JJ40657), 10.13039/501100001809National Natural Science Foundation of China (grant number 82001634) and the 10.13039/501100002858China Postdoctoral Science Foundation (grand number 2019M661521).

## Availability of data and materials

The original contributions presented in the study are included in the article/Supplementary material, further inquiries can be directed to the corresponding author.

## CRediT authorship contribution statement

**Chuan Huang:** Writing – review & editing, Writing – original draft, Funding acquisition, Formal analysis, Data curation, Conceptualization. **Xi-Ren Ji:** Writing – review & editing, Methodology. **Zeng-Hui Huang:** Methodology, Data curation. **Rui-Jun Wang:** Methodology, Data curation. **Li-Qing Fan:** Conceptualization. **Wen-Bing Zhu:** Software, Resources, Conceptualization. **Qiang Luo:** Visualization. **Qing-Li:** Writing – review & editing, Writing – original draft.

## Declaration of competing interest

The authors declare that they have no known competing financial interests or personal relationships that could have appeared to influence the work reported in this paper.
