# Supplementary figures and images for: Global status of research on fertility preservation in male patients with cancer: A bibliometric and visual analysis
Source: Heliyon. 2024 Jun 25;10(13):e33621. doi: 10.1016/j.heliyon.2024.e33621 (PMC11260990; doi:10.1016/j.heliyon.2024.e33621)

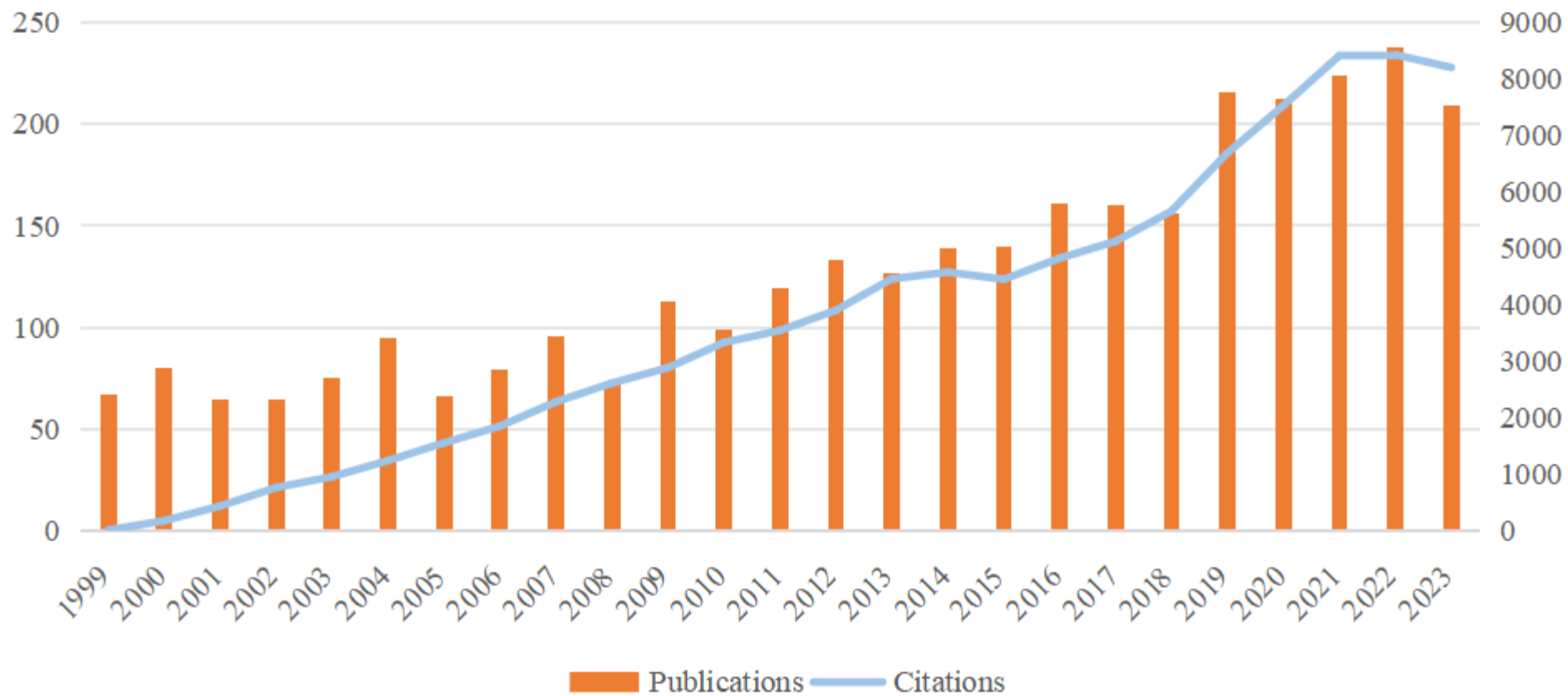

Supplement: Multimedia component 1 [file mmc1.pdf]

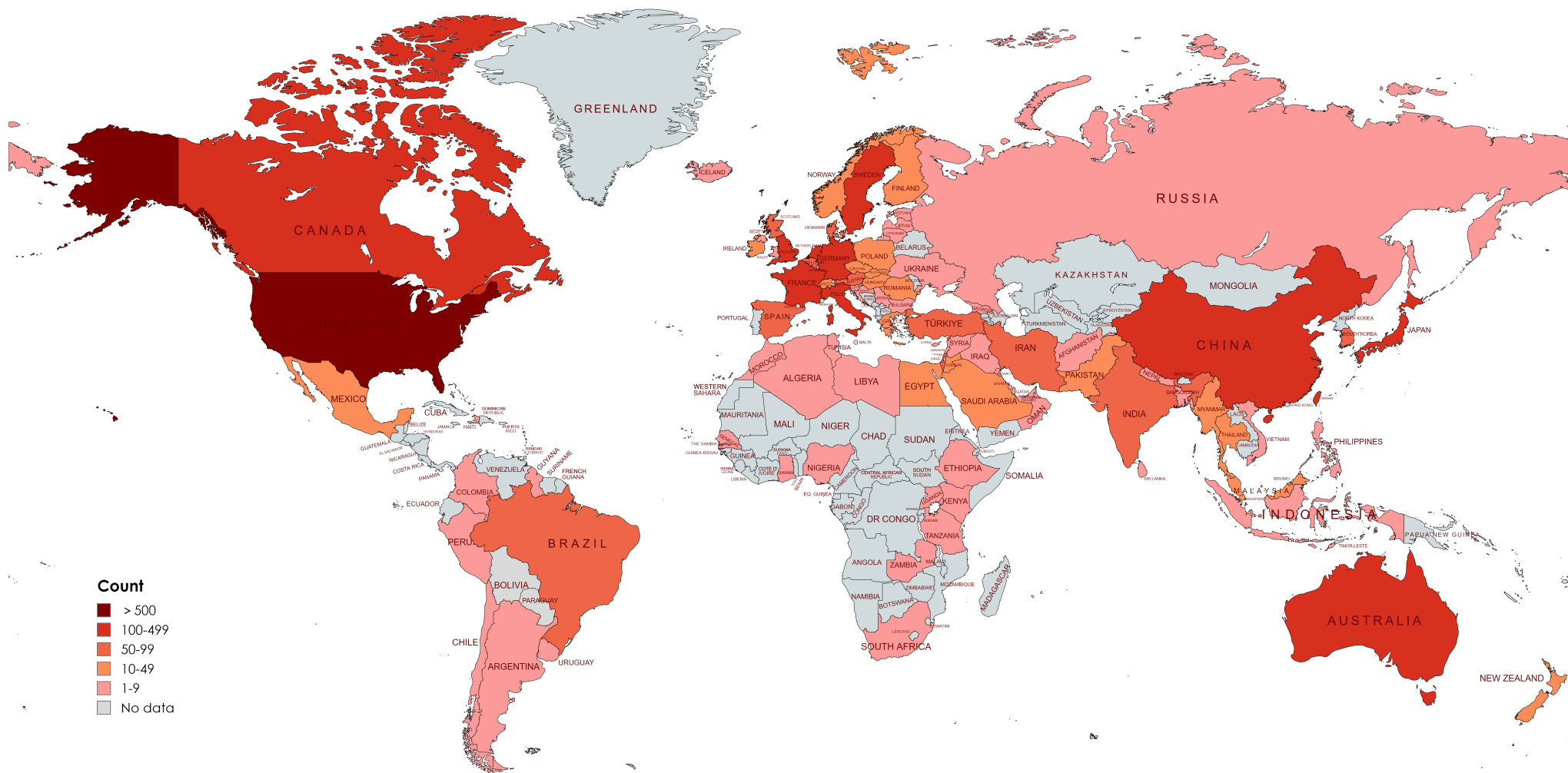

Supplement: Multimedia component 2 [file mmc2.pdf]
