# Supplementary material for: Global status of research on fertility preservation in male patients with cancer: A bibliometric and visual analysis
Source: Heliyon. 2024 Jun 25;10(13):e33621. doi: 10.1016/j.heliyon.2024.e33621 (PMC11260990; doi:10.1016/j.heliyon.2024.e33621)
Supplement: Multimedia component 3 [file mmc3.docx]

**Table S1 Strategies for literature search on male fertility preservation in cancer patients.**

Databases: Web of Science Core Collection

#1: TS=(freezing) OR TS=(banking) OR TS=(cryostorage) OR TS=(storage) OR TS=(cryopreservation) OR TS=(freeze) OR TS=(fertility preservation) OR TS=(Oncofertility)

#2: TS=(male) OR TS=(man) OR TS=(men)

#3: TS=(cancer) OR TS=(tumor) OR TS=(malignancy) OR TS=(neoplasm)

((#1 AND #2) AND DT=(Article AND Review)) AND LA=(English)

Timespan= From January 1999 to December 2023.

**Table S2 Ranking of authors and co-cited authors according to the number of published articles**

| Rank | Author | Count | h-index | Co-cited Author | Count | h-index |
| --- | --- | --- | --- | --- | --- | --- |
| 1 | Agarwal, Ashok | 23 | 101 | Schover Leslie R | 270 | 31 |
| 2 | Nahata, Leena | 20 | 21 | Lee Stephanie J | 233 | 72 |
| 3 | Klosky, James L | 19 | 31 | Oktay Kutluk | 213 | 62 |
| 4 | Quinn, Gwendolyn P | 18 | 46 | Loren Alison W | 206 | 30 |
| 5 | Mitchell, Rod T | 11 | 34 | Meistrich Marvin L | 197 | 67 |
| 6 | Gerhardt, Cynthia A | 10 | 37 | Agarwal, Ashok | 195 | 101 |

**Table S3 Overview of main clusters cited in the literature.**

| **ClusterID** | **Size** | **Silhouette** | **mean(Year)** | **Label (LLR)** |
| --- | --- | --- | --- | --- |
| 0 | 118 | 0.878 | 2012 | fertility preservation (352.94, 1.0E-4); expert meeting (288.68, 1.0E-4); reproductive material (192.53, 1.0E-4); international recommendation (179.22, 1.0E-4); gonadal function (175.9, 1.0E-4) |
| 1 | 114 | 0.916 | 2017 | testicular tissue banking (310.03, 1.0E-4); testicular tissue (272.65, 1.0E-4); testicular cell (251.95, 1.0E-4); male fertility preservation (251.72, 1.0E-4); recent development (202.24, 1.0E-4) |
| 2 | 109 | 0.908 | 2017 | young adult male (334.7, 1.0E-4); adolescent male (259.79, 1.0E-4); fertility preservation (225.79, 1.0E-4); systematic review (223.26, 1.0E-4); following cancer (172.99, 1.0E-4) |
| 3 | 96 | 0.92 | 2000 | current concept (242.07, 1.0E-4); germ cell transplantation (169.08, 1.0E-4); reproductive potential (152.9, 1.0E-4); glycerol propanediol (144.82, 1.0E-4); human fertility (120.6, 1.0E-4) |
| 4 | 85 | 0.857 | 2004 | male cancer survivor (435.83, 1.0E-4); malignant diseases (162.82, 1.0E-4); fertility problem (156.91, 1.0E-4); gonadal dysfunction (156.91, 1.0E-4); gonadal damage (156.53, 1.0E-4) |
| 5 | 82 | 0.86 | 2009 | cancer diagnosis (338.69, 1.0E-4); patient attitude (175.82, 1.0E-4); psychosocial oncofertility issue (175.7, 1.0E-4); psychological aspect (171.06, 1.0E-4); other life-threatening diseases (171.06, 1.0E-4) |
| 6 | 78 | 0.874 | 2008 | fertility consideration (281.48, 1.0E-4); future parenthood (211.24, 1.0E-4); haemato-oncology patient (160.15, 1.0E-4); ovarian response (152.83, 1.0E-4); consortium-addressing fertility (151.19, 1.0E-4) |
| 7 | 67 | 0.914 | 2016 | transgender individual (328.7, 1.0E-4); transgender adolescent (165.62, 1.0E-4); fertility care (159.01, 1.0E-4); gender-specific aspect (159.01, 1.0E-4); transgender youth-a (146.59, 1.0E-4) |
| 8 | 64 | 0.947 | 1998 | reproduction treatment (259.92, 1.0E-4); clinical aspect (167.18, 1.0E-4); biologic basis (167.18, 1.0E-4); following cancer treatment (150.38, 1.0E-4); male partner (150.38, 1.0E-4) |
| 9 | 59 | 0.893 | 2015 | sperm cryopreservation (407.33, 1.0E-4); testicular cancer (323.09, 1.0E-4); male oncological patient (256.2, 1.0E-4); cecos network (198.52, 1.0E-4); fertility preservation (171.01, 1.0E-4) |
| 10 | 38 | 0.941 | 2009 | testicular tissue cryopreservation (165.46, 1.0E-4); spermatogonial stem cell transplantation (147.62, 1.0E-4); fetal pediatric (135.05, 1.0E-4); human spermatogonial stem cell marker (135.05, 1.0E-4); testicular niche (119.34, 1.0E-4) |
| 11 | 22 | 0.986 | 2005 | male fertility (165.81, 1.0E-4); sperm dna integrity (89.89, 1.0E-4); cryopreservation protocol (68.45, 1.0E-4); sperm quality dna integrity (68.45, 1.0E-4); vivo study (47.72, 1.0E-4) |
| 13 | 14 | 0.99 | 2020 | systemic oncological treatment (64.47, 1.0E-4); young adults-a systematic review (64.47, 1.0E-4); young adult hodgkin lymphoma (57.76, 1.0E-4); reproductive ability (57.76, 1.0E-4); sex difference (45.45, 1.0E-4) |
| 19 | 7 | 0.981 | 1995 | quality (29.94, 1.0E-4); life (29.94, 1.0E-4); salvage cryotherapy (29.94, 1.0E-4); treatment parameter (29.94, 1.0E-4); role (14.91, 0.001) |

**Table S4 Journals that published the most-cited 24 publications.**

| Journal | Total cited | Proportion | IF in 2023 |
| --- | --- | --- | --- |
| Cancer | 1156 | 2.7% | 6.2 |
| Journal of Clinical Oncology | 1033 | 2.4% | 45.3 |
| Human Reproduction | 929 | 2.2% | 6.1 |
| Fertility and Sterility | 921 | 2.2% | 6.7 |
| Journal of Urology | 836 | 2.0% | 6.6 |
| New England Journal of Medicine | 767 | 1.8% | 158.5 |
| Lancet | 685 | 1.6% | 168.9 |
| Urology | 654 | 1.5% | 2.1 |
| Cancer Research | 540 | 1.3% | 11.2 |
| PNAS | 539 | 1.3% | 11.1 |
| Human Reproductio Update | 521 | 1.2% | 13.3 |
| European Urology | 508 | 1.2% | 23.4 |
| Plos One | 493 | 1.2% | 3.7 |
| Nature | 488 | 1.1% | 64.8 |
| JAMA | 480 | 1.1% | 120.7 |
| British Journal Of Cancer | 456 | 1.1% | 8.8 |
| Journal Of Clinical Endocrinology & Metabolism | 441 | 1.0% | 5.8 |
| International Journal Of Cancer | 407 | 1.0% | 6.4 |
| Science | 405 | 0.9% | 56.9 |
| Lancet Oncology | 389 | 0.9% | 51.1 |
| CA: A Cancer Journal for Clinicians | 378 | 0.9% | 254.7 |
| BJU International | 359 | 0.8% | 4.5 |
| Annals Of Oncology | 358 | 0.8% | 50.5 |
| Reproductive Biomedicine Online | 340 | 0.8% | 4.0 |

**Table S5 Top 100 Keywords with the Strongest Citation Bursts.**

| **Keywords** | **Year** | **Strength** | **Begin** | **End** | **1999 - 2023** |
| --- | --- | --- | --- | --- | --- |
| hodgkins disease | 1999 | 18.53 | **1999** | 2010 | ▃▃▃▃▃▃▃▃▃▃▃▃▂▂▂▂▂▂▂▂▂▂▂▂▂ |
| intracytoplasmic sperm injection | 1999 | 12.1 | **1999** | 2010 | ▃▃▃▃▃▃▃▃▃▃▃▃▂▂▂▂▂▂▂▂▂▂▂▂▂ |
| carcinoma | 1999 | 11.19 | **1999** | 2007 | ▃▃▃▃▃▃▃▃▃▂▂▂▂▂▂▂▂▂▂▂▂▂▂▂▂ |
| spermatozoa | 1999 | 9.05 | **1999** | 2008 | ▃▃▃▃▃▃▃▃▃▃▂▂▂▂▂▂▂▂▂▂▂▂▂▂▂ |
| fertility | 1999 | 8.44 | **1999** | 2008 | ▃▃▃▃▃▃▃▃▃▃▂▂▂▂▂▂▂▂▂▂▂▂▂▂▂ |
| antigen | 1999 | 7.07 | **1999** | 2011 | ▃▃▃▃▃▃▃▃▃▃▃▃▃▂▂▂▂▂▂▂▂▂▂▂▂ |
| prostate-specific antigen | 1999 | 6.22 | **1999** | 2004 | ▃▃▃▃▃▃▂▂▂▂▂▂▂▂▂▂▂▂▂▂▂▂▂▂▂ |
| carcinoma in situ | 1999 | 5.53 | **1999** | 2011 | ▃▃▃▃▃▃▃▃▃▃▃▃▃▂▂▂▂▂▂▂▂▂▂▂▂ |
| serum | 1999 | 5.5 | **1999** | 2006 | ▃▃▃▃▃▃▃▃▂▂▂▂▂▂▂▂▂▂▂▂▂▂▂▂▂ |
| neoplasms | 1999 | 5.49 | **1999** | 2012 | ▃▃▃▃▃▃▃▃▃▃▃▃▃▃▂▂▂▂▂▂▂▂▂▂▂ |
| lesions | 1999 | 5.05 | **1999** | 2013 | ▃▃▃▃▃▃▃▃▃▃▃▃▃▃▃▂▂▂▂▂▂▂▂▂▂ |
| prostatic neoplasms | 1999 | 4.94 | **1999** | 2005 | ▃▃▃▃▃▃▃▂▂▂▂▂▂▂▂▂▂▂▂▂▂▂▂▂▂ |
| semen cryopreservation | 1999 | 4.88 | **1999** | 2004 | ▃▃▃▃▃▃▂▂▂▂▂▂▂▂▂▂▂▂▂▂▂▂▂▂▂ |
| fertilization | 1999 | 3.43 | **1999** | 2012 | ▃▃▃▃▃▃▃▃▃▃▃▃▃▃▂▂▂▂▂▂▂▂▂▂▂ |
| in situ hybridization | 1999 | 3.39 | **1999** | 2004 | ▃▃▃▃▃▃▂▂▂▂▂▂▂▂▂▂▂▂▂▂▂▂▂▂▂ |
| assay | 1999 | 3.29 | **1999** | 2005 | ▃▃▃▃▃▃▃▂▂▂▂▂▂▂▂▂▂▂▂▂▂▂▂▂▂ |
| combination chemotherapy | 2000 | 7.45 | **2000** | 2007 | ▂▃▃▃▃▃▃▃▃▂▂▂▂▂▂▂▂▂▂▂▂▂▂▂▂ |
| disease | 2000 | 4.68 | **2000** | 2006 | ▂▃▃▃▃▃▃▃▂▂▂▂▂▂▂▂▂▂▂▂▂▂▂▂▂ |
| germ cell tumors | 2000 | 4.55 | **2000** | 2008 | ▂▃▃▃▃▃▃▃▃▃▂▂▂▂▂▂▂▂▂▂▂▂▂▂▂ |
| lung cancer | 2000 | 3.38 | **2000** | 2009 | ▂▃▃▃▃▃▃▃▃▃▃▂▂▂▂▂▂▂▂▂▂▂▂▂▂ |
| trial | 2000 | 3.3 | **2000** | 2009 | ▂▃▃▃▃▃▃▃▃▃▃▂▂▂▂▂▂▂▂▂▂▂▂▂▂ |
| in vitro fertilization | 2001 | 6.25 | **2001** | 2010 | ▂▂▃▃▃▃▃▃▃▃▃▃▂▂▂▂▂▂▂▂▂▂▂▂▂ |
| semen | 2001 | 4.82 | **2001** | 2014 | ▂▂▃▃▃▃▃▃▃▃▃▃▃▃▃▃▂▂▂▂▂▂▂▂▂ |
| recurrence | 2001 | 3.89 | **2001** | 2003 | ▂▂▃▃▃▂▂▂▂▂▂▂▂▂▂▂▂▂▂▂▂▂▂▂▂ |
| prognostic factors | 2001 | 3.68 | **2001** | 2004 | ▂▂▃▃▃▃▂▂▂▂▂▂▂▂▂▂▂▂▂▂▂▂▂▂▂ |
| radiation therapy | 2001 | 3.59 | **2001** | 2014 | ▂▂▃▃▃▃▃▃▃▃▃▃▃▃▃▃▂▂▂▂▂▂▂▂▂ |
| childhood | 2001 | 3.4 | **2001** | 2004 | ▂▂▃▃▃▃▂▂▂▂▂▂▂▂▂▂▂▂▂▂▂▂▂▂▂ |
| hyperplasia | 2002 | 4.57 | **2002** | 2007 | ▂▂▂▃▃▃▃▃▃▂▂▂▂▂▂▂▂▂▂▂▂▂▂▂▂ |
| storage | 2002 | 4.39 | **2002** | 2008 | ▂▂▂▃▃▃▃▃▃▃▂▂▂▂▂▂▂▂▂▂▂▂▂▂▂ |
| diagnosis | 1999 | 4.33 | **2002** | 2006 | ▂▂▂▃▃▃▃▃▂▂▂▂▂▂▂▂▂▂▂▂▂▂▂▂▂ |
| population | 2002 | 3.4 | **2002** | 2007 | ▂▂▂▃▃▃▃▃▃▂▂▂▂▂▂▂▂▂▂▂▂▂▂▂▂ |
| biopsy | 2000 | 5.64 | **2003** | 2010 | ▂▂▂▂▃▃▃▃▃▃▃▃▂▂▂▂▂▂▂▂▂▂▂▂▂ |
| tumors | 1999 | 4.39 | **2003** | 2006 | ▂▂▂▂▃▃▃▃▂▂▂▂▂▂▂▂▂▂▂▂▂▂▂▂▂ |
| cytology | 2003 | 4.13 | **2003** | 2007 | ▂▂▂▂▃▃▃▃▃▂▂▂▂▂▂▂▂▂▂▂▂▂▂▂▂ |
| hepatocellular carcinoma | 2003 | 3.82 | **2003** | 2007 | ▂▂▂▂▃▃▃▃▃▂▂▂▂▂▂▂▂▂▂▂▂▂▂▂▂ |
| accuracy | 2003 | 3.71 | **2003** | 2008 | ▂▂▂▂▃▃▃▃▃▃▂▂▂▂▂▂▂▂▂▂▂▂▂▂▂ |
| ovarian function | 2004 | 3.52 | **2004** | 2008 | ▂▂▂▂▂▃▃▃▃▃▂▂▂▂▂▂▂▂▂▂▂▂▂▂▂ |
| bone marrow transplantation | 2002 | 5.27 | **2005** | 2012 | ▂▂▂▂▂▂▃▃▃▃▃▃▃▃▂▂▂▂▂▂▂▂▂▂▂ |
| plasma | 2005 | 4.39 | **2005** | 2010 | ▂▂▂▂▂▂▃▃▃▃▃▃▂▂▂▂▂▂▂▂▂▂▂▂▂ |
| frozen section | 2003 | 3.71 | **2005** | 2010 | ▂▂▂▂▂▂▃▃▃▃▃▃▂▂▂▂▂▂▂▂▂▂▂▂▂ |
| total body irradiation | 2005 | 3.59 | **2005** | 2011 | ▂▂▂▂▂▂▃▃▃▃▃▃▃▂▂▂▂▂▂▂▂▂▂▂▂ |
| tumor necrosis factor | 2006 | 4.87 | **2006** | 2013 | ▂▂▂▂▂▂▂▃▃▃▃▃▃▃▃▂▂▂▂▂▂▂▂▂▂ |
| damage | 2006 | 3.46 | **2006** | 2009 | ▂▂▂▂▂▂▂▃▃▃▃▂▂▂▂▂▂▂▂▂▂▂▂▂▂ |
| fine needle aspiration | 2000 | 4.24 | **2007** | 2012 | ▂▂▂▂▂▂▂▂▃▃▃▃▃▃▂▂▂▂▂▂▂▂▂▂▂ |
| frozen section analysis | 2007 | 3.86 | **2007** | 2011 | ▂▂▂▂▂▂▂▂▃▃▃▃▃▂▂▂▂▂▂▂▂▂▂▂▂ |
| cancer survivors | 2007 | 3.44 | **2007** | 2016 | ▂▂▂▂▂▂▂▂▃▃▃▃▃▃▃▃▃▃▂▂▂▂▂▂▂ |
| identification | 2004 | 3.5 | **2009** | 2014 | ▂▂▂▂▂▂▂▂▂▂▃▃▃▃▃▃▂▂▂▂▂▂▂▂▂ |
| cryopreserved ovarian tissue | 2006 | 3.45 | **2010** | 2016 | ▂▂▂▂▂▂▂▂▂▂▂▃▃▃▃▃▃▃▂▂▂▂▂▂▂ |
| benign prostatic hyperplasia | 2011 | 8.74 | **2011** | 2015 | ▂▂▂▂▂▂▂▂▂▂▂▂▃▃▃▃▃▂▂▂▂▂▂▂▂ |
| survivors | 2006 | 5.77 | **2011** | 2015 | ▂▂▂▂▂▂▂▂▂▂▂▂▃▃▃▃▃▂▂▂▂▂▂▂▂ |
| young women | 2008 | 5.13 | **2012** | 2015 | ▂▂▂▂▂▂▂▂▂▂▂▂▂▃▃▃▃▂▂▂▂▂▂▂▂ |
| parameters | 2006 | 4.63 | **2012** | 2015 | ▂▂▂▂▂▂▂▂▂▂▂▂▂▃▃▃▃▂▂▂▂▂▂▂▂ |
| risk factors | 2005 | 4.08 | **2012** | 2015 | ▂▂▂▂▂▂▂▂▂▂▂▂▂▃▃▃▃▂▂▂▂▂▂▂▂ |
| adolescents | 2008 | 6.92 | **2013** | 2018 | ▂▂▂▂▂▂▂▂▂▂▂▂▂▂▃▃▃▃▃▃▂▂▂▂▂ |
| mouse | 2003 | 5.98 | **2013** | 2016 | ▂▂▂▂▂▂▂▂▂▂▂▂▂▂▃▃▃▃▂▂▂▂▂▂▂ |
| decision making | 2013 | 5.64 | **2013** | 2019 | ▂▂▂▂▂▂▂▂▂▂▂▂▂▂▃▃▃▃▃▃▃▂▂▂▂ |
| vitrification | 2013 | 5.01 | **2013** | 2017 | ▂▂▂▂▂▂▂▂▂▂▂▂▂▂▃▃▃▃▃▂▂▂▂▂▂ |
| follow up | 1999 | 4.78 | **2013** | 2018 | ▂▂▂▂▂▂▂▂▂▂▂▂▂▂▃▃▃▃▃▃▂▂▂▂▂ |
| metabolic syndrome | 2008 | 3.64 | **2013** | 2015 | ▂▂▂▂▂▂▂▂▂▂▂▂▂▂▃▃▃▂▂▂▂▂▂▂▂ |
| prevalence | 2000 | 5.21 | **2014** | 2018 | ▂▂▂▂▂▂▂▂▂▂▂▂▂▂▂▃▃▃▃▃▂▂▂▂▂ |
| gastric cancer | 1999 | 3.74 | **2014** | 2016 | ▂▂▂▂▂▂▂▂▂▂▂▂▂▂▂▃▃▃▂▂▂▂▂▂▂ |
| reproductive health | 2014 | 3.71 | **2014** | 2019 | ▂▂▂▂▂▂▂▂▂▂▂▂▂▂▂▃▃▃▃▃▃▂▂▂▂ |
| american society | 2014 | 11.05 | **2015** | 2020 | ▂▂▂▂▂▂▂▂▂▂▂▂▂▂▂▂▃▃▃▃▃▃▂▂▂ |
| ovarian tissue cryopreservation | 2009 | 6.28 | **2016** | 2020 | ▂▂▂▂▂▂▂▂▂▂▂▂▂▂▂▂▂▃▃▃▃▃▂▂▂ |
| knowledge | 2016 | 5.91 | **2016** | 2017 | ▂▂▂▂▂▂▂▂▂▂▂▂▂▂▂▂▂▃▃▂▂▂▂▂▂ |
| care | 2014 | 5.53 | **2016** | 2021 | ▂▂▂▂▂▂▂▂▂▂▂▂▂▂▂▂▂▃▃▃▃▃▃▂▂ |
| inflammation | 2009 | 5.18 | **2016** | 2020 | ▂▂▂▂▂▂▂▂▂▂▂▂▂▂▂▂▂▃▃▃▃▃▂▂▂ |
| united states | 2014 | 4.86 | **2016** | 2023 | ▂▂▂▂▂▂▂▂▂▂▂▂▂▂▂▂▂▃▃▃▃▃▃▃▃ |
| oncology | 2016 | 4.86 | **2016** | 2017 | ▂▂▂▂▂▂▂▂▂▂▂▂▂▂▂▂▂▃▃▂▂▂▂▂▂ |
| metaanalysis | 2008 | 4.36 | **2016** | 2020 | ▂▂▂▂▂▂▂▂▂▂▂▂▂▂▂▂▂▃▃▃▃▃▂▂▂ |
| progression | 2003 | 3.71 | **2016** | 2017 | ▂▂▂▂▂▂▂▂▂▂▂▂▂▂▂▂▂▃▃▂▂▂▂▂▂ |
| motility | 2016 | 3.59 | **2016** | 2019 | ▂▂▂▂▂▂▂▂▂▂▂▂▂▂▂▂▂▃▃▃▃▂▂▂▂ |
| adolescent | 2009 | 7.72 | **2017** | 2023 | ▂▂▂▂▂▂▂▂▂▂▂▂▂▂▂▂▂▂▃▃▃▃▃▃▃ |
| young adults | 2017 | 7.62 | **2017** | 2019 | ▂▂▂▂▂▂▂▂▂▂▂▂▂▂▂▂▂▂▃▃▃▂▂▂▂ |
| semen parameters | 2017 | 3.78 | **2017** | 2018 | ▂▂▂▂▂▂▂▂▂▂▂▂▂▂▂▂▂▂▃▃▂▂▂▂▂ |
| adult survivors | 2017 | 3.5 | **2017** | 2020 | ▂▂▂▂▂▂▂▂▂▂▂▂▂▂▂▂▂▂▃▃▃▃▂▂▂ |
| issue | 2003 | 3.46 | **2017** | 2019 | ▂▂▂▂▂▂▂▂▂▂▂▂▂▂▂▂▂▂▃▃▃▂▂▂▂ |
| experiences | 2013 | 4.59 | **2018** | 2020 | ▂▂▂▂▂▂▂▂▂▂▂▂▂▂▂▂▂▂▂▃▃▃▂▂▂ |
| proliferation | 2018 | 3.49 | **2018** | 2021 | ▂▂▂▂▂▂▂▂▂▂▂▂▂▂▂▂▂▂▂▃▃▃▃▂▂ |
| mutations | 2006 | 3.36 | **2018** | 2021 | ▂▂▂▂▂▂▂▂▂▂▂▂▂▂▂▂▂▂▂▃▃▃▃▂▂ |
| case report | 2019 | 11.2 | **2019** | 2023 | ▂▂▂▂▂▂▂▂▂▂▂▂▂▂▂▂▂▂▂▂▃▃▃▃▃ |
| testicular tissue cryopreservation | 2019 | 6.07 | **2019** | 2020 | ▂▂▂▂▂▂▂▂▂▂▂▂▂▂▂▂▂▂▂▂▃▃▂▂▂ |
| sperm | 2000 | 4.99 | **2019** | 2023 | ▂▂▂▂▂▂▂▂▂▂▂▂▂▂▂▂▂▂▂▂▃▃▃▃▃ |
| stem cell transplantation | 2019 | 4.59 | **2019** | 2023 | ▂▂▂▂▂▂▂▂▂▂▂▂▂▂▂▂▂▂▂▂▃▃▃▃▃ |
| obesity | 2019 | 4.19 | **2019** | 2023 | ▂▂▂▂▂▂▂▂▂▂▂▂▂▂▂▂▂▂▂▂▃▃▃▃▃ |
| pediatric cancer | 2019 | 3.76 | **2019** | 2023 | ▂▂▂▂▂▂▂▂▂▂▂▂▂▂▂▂▂▂▂▂▃▃▃▃▃ |
| stimulation | 2019 | 3.31 | **2019** | 2020 | ▂▂▂▂▂▂▂▂▂▂▂▂▂▂▂▂▂▂▂▂▃▃▂▂▂ |
| fertility preservation | 2003 | 5.58 | **2020** | 2023 | ▂▂▂▂▂▂▂▂▂▂▂▂▂▂▂▂▂▂▂▂▂▃▃▃▃ |
| sperm cryopreservation | 2000 | 5.57 | **2020** | 2021 | ▂▂▂▂▂▂▂▂▂▂▂▂▂▂▂▂▂▂▂▂▂▃▃▂▂ |
| epidemiology | 2001 | 5.41 | **2020** | 2023 | ▂▂▂▂▂▂▂▂▂▂▂▂▂▂▂▂▂▂▂▂▂▃▃▃▃ |
| health | 1999 | 4.63 | **2020** | 2023 | ▂▂▂▂▂▂▂▂▂▂▂▂▂▂▂▂▂▂▂▂▂▃▃▃▃ |
| alkylating agent exposure | 2018 | 3.71 | **2020** | 2023 | ▂▂▂▂▂▂▂▂▂▂▂▂▂▂▂▂▂▂▂▂▂▃▃▃▃ |
| male fertility | 2010 | 3.71 | **2020** | 2023 | ▂▂▂▂▂▂▂▂▂▂▂▂▂▂▂▂▂▂▂▂▂▃▃▃▃ |
| recommendations | 2015 | 5.16 | **2021** | 2023 | ▂▂▂▂▂▂▂▂▂▂▂▂▂▂▂▂▂▂▂▂▂▂▃▃▃ |
| mechanisms | 2006 | 4.5 | **2021** | 2023 | ▂▂▂▂▂▂▂▂▂▂▂▂▂▂▂▂▂▂▂▂▂▂▃▃▃ |
| reconstruction | 2021 | 3.92 | **2021** | 2023 | ▂▂▂▂▂▂▂▂▂▂▂▂▂▂▂▂▂▂▂▂▂▂▃▃▃ |
| benign | 2021 | 3.92 | **2021** | 2023 | ▂▂▂▂▂▂▂▂▂▂▂▂▂▂▂▂▂▂▂▂▂▂▃▃▃ |
| in vitro spermatogenesis | 2021 | 3.92 | **2021** | 2023 | ▂▂▂▂▂▂▂▂▂▂▂▂▂▂▂▂▂▂▂▂▂▂▃▃▃ |
| tissue | 2003 | 3.74 | **2021** | 2023 | ▂▂▂▂▂▂▂▂▂▂▂▂▂▂▂▂▂▂▂▂▂▂▃▃▃ |
| radical orchiectomy | 2021 | 3.35 | **2021** | 2023 | ▂▂▂▂▂▂▂▂▂▂▂▂▂▂▂▂▂▂▂▂▂▂▃▃▃ |

**Table S5 Top 399 Cited Journals with the Strongest Citation Bursts**

| **Cited Journals** | **Year** | **Strength** | **Begin** | **End** | **1999 - 2023** |
| --- | --- | --- | --- | --- | --- |
| BRIT J UROL | 1999 | 37.39 | **1999** | 2007 | ▃▃▃▃▃▃▃▃▃▂▂▂▂▂▂▂▂▂▂▂▂▂▂▂▂ |
| CANCER RES | 1999 | 34.79 | **1999** | 2007 | ▃▃▃▃▃▃▃▃▃▂▂▂▂▂▂▂▂▂▂▂▂▂▂▂▂ |
| J NATL CANCER I | 1999 | 29.87 | **1999** | 2011 | ▃▃▃▃▃▃▃▃▃▃▃▃▃▂▂▂▂▂▂▂▂▂▂▂▂ |
| ARCH SURG-CHICAGO | 1999 | 23.44 | **1999** | 2012 | ▃▃▃▃▃▃▃▃▃▃▃▃▃▃▂▂▂▂▂▂▂▂▂▂▂ |
| AM J PATHOL | 1999 | 20.84 | **1999** | 2012 | ▃▃▃▃▃▃▃▃▃▃▃▃▃▃▂▂▂▂▂▂▂▂▂▂▂ |
| CLIN CHEM | 1999 | 20.52 | **1999** | 2011 | ▃▃▃▃▃▃▃▃▃▃▃▃▃▂▂▂▂▂▂▂▂▂▂▂▂ |
| BRIT MED J | 1999 | 18.62 | **1999** | 2013 | ▃▃▃▃▃▃▃▃▃▃▃▃▃▃▃▂▂▂▂▂▂▂▂▂▂ |
| AM J CLIN PATHOL | 1999 | 16.83 | **1999** | 2009 | ▃▃▃▃▃▃▃▃▃▃▃▂▂▂▂▂▂▂▂▂▂▂▂▂▂ |
| AM J MED | 1999 | 15.38 | **1999** | 2011 | ▃▃▃▃▃▃▃▃▃▃▃▃▃▂▂▂▂▂▂▂▂▂▂▂▂ |
| BRIT J SURG | 1999 | 14.18 | **1999** | 2009 | ▃▃▃▃▃▃▃▃▃▃▃▂▂▂▂▂▂▂▂▂▂▂▂▂▂ |
| UROL CLIN N AM | 1999 | 14.01 | **1999** | 2007 | ▃▃▃▃▃▃▃▃▃▂▂▂▂▂▂▂▂▂▂▂▂▂▂▂▂ |
| ANN SURG | 1999 | 13.31 | **1999** | 2007 | ▃▃▃▃▃▃▃▃▃▂▂▂▂▂▂▂▂▂▂▂▂▂▂▂▂ |
| HUM PATHOL | 1999 | 12.35 | **1999** | 2007 | ▃▃▃▃▃▃▃▃▃▂▂▂▂▂▂▂▂▂▂▂▂▂▂▂▂ |
| LAB INVEST | 1999 | 12.25 | **1999** | 2007 | ▃▃▃▃▃▃▃▃▃▂▂▂▂▂▂▂▂▂▂▂▂▂▂▂▂ |
| BIOCHIM BIOPHYS ACTA | 1999 | 11 | **1999** | 2007 | ▃▃▃▃▃▃▃▃▃▂▂▂▂▂▂▂▂▂▂▂▂▂▂▂▂ |
| JAMA-J AM MED ASSOC | 1999 | 10.63 | **1999** | 2003 | ▃▃▃▃▃▂▂▂▂▂▂▂▂▂▂▂▂▂▂▂▂▂▂▂▂ |
| AM J HUM GENET | 1999 | 10.48 | **1999** | 2009 | ▃▃▃▃▃▃▃▃▃▃▃▂▂▂▂▂▂▂▂▂▂▂▂▂▂ |
| CANCER GENET CYTOGEN | 1999 | 10.38 | **1999** | 2008 | ▃▃▃▃▃▃▃▃▃▃▂▂▂▂▂▂▂▂▂▂▂▂▂▂▂ |
| CARCINOGENESIS | 1999 | 10.16 | **1999** | 2012 | ▃▃▃▃▃▃▃▃▃▃▃▃▃▃▂▂▂▂▂▂▂▂▂▂▂ |
| MUTAT RES | 1999 | 9.6 | **1999** | 2008 | ▃▃▃▃▃▃▃▃▃▃▂▂▂▂▂▂▂▂▂▂▂▂▂▂▂ |
| PROSTATE | 1999 | 9.09 | **1999** | 2005 | ▃▃▃▃▃▃▃▂▂▂▂▂▂▂▂▂▂▂▂▂▂▂▂▂▂ |
| AM J SURG | 1999 | 8.8 | **1999** | 2010 | ▃▃▃▃▃▃▃▃▃▃▃▃▂▂▂▂▂▂▂▂▂▂▂▂▂ |
| ANN INTERN MED | 1999 | 8.52 | **1999** | 2005 | ▃▃▃▃▃▃▃▂▂▂▂▂▂▂▂▂▂▂▂▂▂▂▂▂▂ |
| ONCOLOGY | 1999 | 8.47 | **1999** | 2005 | ▃▃▃▃▃▃▃▂▂▂▂▂▂▂▂▂▂▂▂▂▂▂▂▂▂ |
| CHEST | 1999 | 8.01 | **1999** | 2013 | ▃▃▃▃▃▃▃▃▃▃▃▃▃▃▃▂▂▂▂▂▂▂▂▂▂ |
| AM J EPIDEMIOL | 1999 | 7.92 | **1999** | 2013 | ▃▃▃▃▃▃▃▃▃▃▃▃▃▃▃▂▂▂▂▂▂▂▂▂▂ |
| CANCER LETT | 1999 | 7.74 | **1999** | 2007 | ▃▃▃▃▃▃▃▃▃▂▂▂▂▂▂▂▂▂▂▂▂▂▂▂▂ |
| AM SURGEON | 1999 | 7.74 | **1999** | 2012 | ▃▃▃▃▃▃▃▃▃▃▃▃▃▃▂▂▂▂▂▂▂▂▂▂▂ |
| HUM GENET | 1999 | 7.08 | **1999** | 2008 | ▃▃▃▃▃▃▃▃▃▃▂▂▂▂▂▂▂▂▂▂▂▂▂▂▂ |
| JOURNAL OF UROLOGY | 1999 | 7 | **1999** | 2001 | ▃▃▃▂▂▂▂▂▂▂▂▂▂▂▂▂▂▂▂▂▂▂▂▂▂ |
| J PATHOL | 1999 | 6.85 | **1999** | 2004 | ▃▃▃▃▃▃▂▂▂▂▂▂▂▂▂▂▂▂▂▂▂▂▂▂▂ |
| INT J EPIDEMIOL | 1999 | 6.83 | **1999** | 2006 | ▃▃▃▃▃▃▃▃▂▂▂▂▂▂▂▂▂▂▂▂▂▂▂▂▂ |
| AM J PHYSIOL | 1999 | 6.74 | **1999** | 2010 | ▃▃▃▃▃▃▃▃▃▃▃▃▂▂▂▂▂▂▂▂▂▂▂▂▂ |
| CANCER | 1999 | 6.46 | **1999** | 2000 | ▃▃▂▂▂▂▂▂▂▂▂▂▂▂▂▂▂▂▂▂▂▂▂▂▂ |
| BRIT J OBSTET GYNAEC | 1999 | 6.27 | **1999** | 2003 | ▃▃▃▃▃▂▂▂▂▂▂▂▂▂▂▂▂▂▂▂▂▂▂▂▂ |
| J UROLOGY | 1999 | 5.96 | **1999** | 2003 | ▃▃▃▃▃▂▂▂▂▂▂▂▂▂▂▂▂▂▂▂▂▂▂▂▂ |
| FEBS LETT | 1999 | 5.75 | **1999** | 2006 | ▃▃▃▃▃▃▃▃▂▂▂▂▂▂▂▂▂▂▂▂▂▂▂▂▂ |
| PATHOL RES PRACT | 1999 | 5.69 | **1999** | 2004 | ▃▃▃▃▃▃▂▂▂▂▂▂▂▂▂▂▂▂▂▂▂▂▂▂▂ |
| AM J CLIN NUTR | 1999 | 5.35 | **1999** | 2011 | ▃▃▃▃▃▃▃▃▃▃▃▃▃▂▂▂▂▂▂▂▂▂▂▂▂ |
| ANN MED | 1999 | 5.32 | **1999** | 2015 | ▃▃▃▃▃▃▃▃▃▃▃▃▃▃▃▃▃▂▂▂▂▂▂▂▂ |
| ENVIRON MOL MUTAGEN | 1999 | 5.21 | **1999** | 2004 | ▃▃▃▃▃▃▂▂▂▂▂▂▂▂▂▂▂▂▂▂▂▂▂▂▂ |
| ACTA PATHOL JAPON | 1999 | 4.97 | **1999** | 2002 | ▃▃▃▃▂▂▂▂▂▂▂▂▂▂▂▂▂▂▂▂▂▂▂▂▂ |
| SCIENCE | 1999 | 4.86 | **1999** | 2003 | ▃▃▃▃▃▂▂▂▂▂▂▂▂▂▂▂▂▂▂▂▂▂▂▂▂ |
| P NATL ACAD SCI USA | 1999 | 4.84 | **1999** | 2001 | ▃▃▃▂▂▂▂▂▂▂▂▂▂▂▂▂▂▂▂▂▂▂▂▂▂ |
| IARC SCI PUBL | 1999 | 4.71 | **1999** | 2007 | ▃▃▃▃▃▃▃▃▃▂▂▂▂▂▂▂▂▂▂▂▂▂▂▂▂ |
| ARCH OPHTHALMOL-CHIC | 1999 | 4.63 | **1999** | 2008 | ▃▃▃▃▃▃▃▃▃▃▂▂▂▂▂▂▂▂▂▂▂▂▂▂▂ |
| VIRCHOWS ARCH A | 1999 | 4.59 | **1999** | 2000 | ▃▃▂▂▂▂▂▂▂▂▂▂▂▂▂▂▂▂▂▂▂▂▂▂▂ |
| J HISTOCHEM CYTOCHEM | 1999 | 4.53 | **1999** | 2012 | ▃▃▃▃▃▃▃▃▃▃▃▃▃▃▂▂▂▂▂▂▂▂▂▂▂ |
| INVEST UROL | 1999 | 4.46 | **1999** | 2001 | ▃▃▃▂▂▂▂▂▂▂▂▂▂▂▂▂▂▂▂▂▂▂▂▂▂ |
| BIOCHEMISTRY-US | 1999 | 4.35 | **1999** | 2006 | ▃▃▃▃▃▃▃▃▂▂▂▂▂▂▂▂▂▂▂▂▂▂▂▂▂ |
| SEMIN DIAGN PATHOL | 1999 | 3.94 | **1999** | 2000 | ▃▃▂▂▂▂▂▂▂▂▂▂▂▂▂▂▂▂▂▂▂▂▂▂▂ |
| JPN J CANCER RES | 1999 | 3.94 | **1999** | 2000 | ▃▃▂▂▂▂▂▂▂▂▂▂▂▂▂▂▂▂▂▂▂▂▂▂▂ |
| KIDNEY INT | 1999 | 3.66 | **1999** | 2007 | ▃▃▃▃▃▃▃▃▃▂▂▂▂▂▂▂▂▂▂▂▂▂▂▂▂ |
| DIGEST DIS SCI | 1999 | 3.61 | **1999** | 2003 | ▃▃▃▃▃▂▂▂▂▂▂▂▂▂▂▂▂▂▂▂▂▂▂▂▂ |
| ACTA ENDOCRINOL-COP | 1999 | 3.28 | **1999** | 2000 | ▃▃▂▂▂▂▂▂▂▂▂▂▂▂▂▂▂▂▂▂▂▂▂▂▂ |
| SURGERY | 2000 | 23.86 | **2000** | 2010 | ▂▃▃▃▃▃▃▃▃▃▃▃▂▂▂▂▂▂▂▂▂▂▂▂▂ |
| MED PEDIATR ONCOL | 1999 | 11.31 | **2000** | 2012 | ▂▃▃▃▃▃▃▃▃▃▃▃▃▃▂▂▂▂▂▂▂▂▂▂▂ |
| EUR J CANCER CLIN ON | 2000 | 9.82 | **2000** | 2010 | ▂▃▃▃▃▃▃▃▃▃▃▃▂▂▂▂▂▂▂▂▂▂▂▂▂ |
| SURG TODAY | 2000 | 9.46 | **2000** | 2012 | ▂▃▃▃▃▃▃▃▃▃▃▃▃▃▂▂▂▂▂▂▂▂▂▂▂ |
| GASTROENTEROLOGY | 1999 | 8.35 | **2000** | 2004 | ▂▃▃▃▃▃▂▂▂▂▂▂▂▂▂▂▂▂▂▂▂▂▂▂▂ |
| CANCER CHEMOTH PHARM | 2000 | 6.98 | **2000** | 2011 | ▂▃▃▃▃▃▃▃▃▃▃▃▃▂▂▂▂▂▂▂▂▂▂▂▂ |
| AM J GASTROENTEROL | 2000 | 6.73 | **2000** | 2007 | ▂▃▃▃▃▃▃▃▃▂▂▂▂▂▂▂▂▂▂▂▂▂▂▂▂ |
| AUST NZ J SURG | 2000 | 6.39 | **2000** | 2005 | ▂▃▃▃▃▃▃▂▂▂▂▂▂▂▂▂▂▂▂▂▂▂▂▂▂ |
| NEW ENGL J MED | 1999 | 5.67 | **2000** | 2004 | ▂▃▃▃▃▃▂▂▂▂▂▂▂▂▂▂▂▂▂▂▂▂▂▂▂ |
| SEMIN SURG ONCOL | 2000 | 5.36 | **2000** | 2004 | ▂▃▃▃▃▃▂▂▂▂▂▂▂▂▂▂▂▂▂▂▂▂▂▂▂ |
| ANN HEMATOL | 2000 | 5.19 | **2000** | 2004 | ▂▃▃▃▃▃▂▂▂▂▂▂▂▂▂▂▂▂▂▂▂▂▂▂▂ |
| J CELL BIOL | 2000 | 4.96 | **2000** | 2001 | ▂▃▃▂▂▂▂▂▂▂▂▂▂▂▂▂▂▂▂▂▂▂▂▂▂ |
| NAT GENET | 2000 | 4.79 | **2000** | 2003 | ▂▃▃▃▃▂▂▂▂▂▂▂▂▂▂▂▂▂▂▂▂▂▂▂▂ |
| BRIT J RADIOL | 2000 | 4.64 | **2000** | 2006 | ▂▃▃▃▃▃▃▃▂▂▂▂▂▂▂▂▂▂▂▂▂▂▂▂▂ |
| J EXP MED | 2000 | 4.57 | **2000** | 2011 | ▂▃▃▃▃▃▃▃▃▃▃▃▃▂▂▂▂▂▂▂▂▂▂▂▂ |
| BLOOD REV | 2000 | 4.32 | **2000** | 2011 | ▂▃▃▃▃▃▃▃▃▃▃▃▃▂▂▂▂▂▂▂▂▂▂▂▂ |
| BRAIN RES | 2000 | 4.19 | **2000** | 2010 | ▂▃▃▃▃▃▃▃▃▃▃▃▂▂▂▂▂▂▂▂▂▂▂▂▂ |
| GUT | 2000 | 3.73 | **2000** | 2003 | ▂▃▃▃▃▂▂▂▂▂▂▂▂▂▂▂▂▂▂▂▂▂▂▂▂ |
| ARCH OTOLARYNGOL | 2001 | 9.1 | **2001** | 2013 | ▂▂▃▃▃▃▃▃▃▃▃▃▃▃▃▂▂▂▂▂▂▂▂▂▂ |
| EUR J SURG ONCOL | 2001 | 8.73 | **2001** | 2009 | ▂▂▃▃▃▃▃▃▃▃▃▂▂▂▂▂▂▂▂▂▂▂▂▂▂ |
| PROG CLIN BIOL RES | 2001 | 8.32 | **2001** | 2003 | ▂▂▃▃▃▂▂▂▂▂▂▂▂▂▂▂▂▂▂▂▂▂▂▂▂ |
| HEPATO-GASTROENTEROL | 2001 | 8.28 | **2001** | 2012 | ▂▂▃▃▃▃▃▃▃▃▃▃▃▃▂▂▂▂▂▂▂▂▂▂▂ |
| ENDOCRINOLOGY | 1999 | 8.21 | **2001** | 2005 | ▂▂▃▃▃▃▃▂▂▂▂▂▂▂▂▂▂▂▂▂▂▂▂▂▂ |
| LANCET | 1999 | 8.02 | **2001** | 2005 | ▂▂▃▃▃▃▃▂▂▂▂▂▂▂▂▂▂▂▂▂▂▂▂▂▂ |
| J NUCL MED | 2001 | 7.24 | **2001** | 2006 | ▂▂▃▃▃▃▃▃▂▂▂▂▂▂▂▂▂▂▂▂▂▂▂▂▂ |
| ANAL BIOCHEM | 2001 | 6.5 | **2001** | 2009 | ▂▂▃▃▃▃▃▃▃▃▃▂▂▂▂▂▂▂▂▂▂▂▂▂▂ |
| RADIOLOGY | 1999 | 6.48 | **2001** | 2007 | ▂▂▃▃▃▃▃▃▃▂▂▂▂▂▂▂▂▂▂▂▂▂▂▂▂ |
| NAT MED | 2000 | 5.82 | **2001** | 2006 | ▂▂▃▃▃▃▃▃▂▂▂▂▂▂▂▂▂▂▂▂▂▂▂▂▂ |
| AM J IND MED | 2001 | 4.81 | **2001** | 2005 | ▂▂▃▃▃▃▃▂▂▂▂▂▂▂▂▂▂▂▂▂▂▂▂▂▂ |
| EXP CELL RES | 2001 | 4.75 | **2001** | 2009 | ▂▂▃▃▃▃▃▃▃▃▃▂▂▂▂▂▂▂▂▂▂▂▂▂▂ |
| CIRCULATION | 1999 | 3.57 | **2001** | 2007 | ▂▂▃▃▃▃▃▃▃▂▂▂▂▂▂▂▂▂▂▂▂▂▂▂▂ |
| ANNU REV BIOCHEM | 2001 | 3.34 | **2001** | 2014 | ▂▂▃▃▃▃▃▃▃▃▃▃▃▃▃▃▂▂▂▂▂▂▂▂▂ |
| WORLD J SURG | 2000 | 12.17 | **2002** | 2012 | ▂▂▂▃▃▃▃▃▃▃▃▃▃▃▂▂▂▂▂▂▂▂▂▂▂ |
| NUTR CANCER | 1999 | 10.35 | **2002** | 2006 | ▂▂▂▃▃▃▃▃▂▂▂▂▂▂▂▂▂▂▂▂▂▂▂▂▂ |
| SCAND J UROL NEPHROL | 2002 | 8.99 | **2002** | 2015 | ▂▂▂▃▃▃▃▃▃▃▃▃▃▃▃▃▃▂▂▂▂▂▂▂▂ |
| ARCH PATHOL LAB MED | 1999 | 8.72 | **2002** | 2010 | ▂▂▂▃▃▃▃▃▃▃▃▃▂▂▂▂▂▂▂▂▂▂▂▂▂ |
| MUTAT RES-FUND MOL M | 2002 | 7.94 | **2002** | 2009 | ▂▂▂▃▃▃▃▃▃▃▃▂▂▂▂▂▂▂▂▂▂▂▂▂▂ |
| CANCER CAUSE CONTROL | 2000 | 7.24 | **2002** | 2006 | ▂▂▂▃▃▃▃▃▂▂▂▂▂▂▂▂▂▂▂▂▂▂▂▂▂ |
| BRIT J NUTR | 2002 | 6.2 | **2002** | 2015 | ▂▂▂▃▃▃▃▃▃▃▃▃▃▃▃▃▃▂▂▂▂▂▂▂▂ |
| GENE CHROMOSOME CANC | 2002 | 6.14 | **2002** | 2011 | ▂▂▂▃▃▃▃▃▃▃▃▃▃▂▂▂▂▂▂▂▂▂▂▂▂ |
| EPIDEMIOLOGY | 2002 | 5.64 | **2002** | 2010 | ▂▂▂▃▃▃▃▃▃▃▃▃▂▂▂▂▂▂▂▂▂▂▂▂▂ |
| UROLOGY | 1999 | 5.52 | **2002** | 2004 | ▂▂▂▃▃▃▂▂▂▂▂▂▂▂▂▂▂▂▂▂▂▂▂▂▂ |
| CAMPBELLS UROLOGY | 2002 | 5.06 | **2002** | 2008 | ▂▂▂▃▃▃▃▃▃▃▂▂▂▂▂▂▂▂▂▂▂▂▂▂▂ |
| J AGR FOOD CHEM | 2002 | 5.03 | **2002** | 2005 | ▂▂▂▃▃▃▃▂▂▂▂▂▂▂▂▂▂▂▂▂▂▂▂▂▂ |
| EUR J CLIN NUTR | 2002 | 4.34 | **2002** | 2015 | ▂▂▂▃▃▃▃▃▃▃▃▃▃▃▃▃▃▂▂▂▂▂▂▂▂ |
| LARYNGOSCOPE | 2002 | 3.95 | **2002** | 2007 | ▂▂▂▃▃▃▃▃▃▂▂▂▂▂▂▂▂▂▂▂▂▂▂▂▂ |
| AM J RESP CRIT CARE | 2002 | 3.94 | **2002** | 2013 | ▂▂▂▃▃▃▃▃▃▃▃▃▃▃▃▂▂▂▂▂▂▂▂▂▂ |
| J LAB CLIN MED | 2002 | 3.73 | **2002** | 2009 | ▂▂▂▃▃▃▃▃▃▃▃▂▂▂▂▂▂▂▂▂▂▂▂▂▂ |
| ANN THORAC SURG | 2001 | 3.72 | **2002** | 2006 | ▂▂▂▃▃▃▃▃▂▂▂▂▂▂▂▂▂▂▂▂▂▂▂▂▂ |
| SCAND J GASTROENTERO | 2002 | 3.6 | **2002** | 2006 | ▂▂▂▃▃▃▃▃▂▂▂▂▂▂▂▂▂▂▂▂▂▂▂▂▂ |
| BRIT MED BULL | 2002 | 3.3 | **2002** | 2003 | ▂▂▂▃▃▂▂▂▂▂▂▂▂▂▂▂▂▂▂▂▂▂▂▂▂ |
| ACTA CYTOL | 1999 | 11.64 | **2003** | 2012 | ▂▂▂▂▃▃▃▃▃▃▃▃▃▃▂▂▂▂▂▂▂▂▂▂▂ |
| NEUROSURGERY | 2003 | 10.07 | **2003** | 2008 | ▂▂▂▂▃▃▃▃▃▃▂▂▂▂▂▂▂▂▂▂▂▂▂▂▂ |
| DIAGN CYTOPATHOL | 2000 | 9.47 | **2003** | 2015 | ▂▂▂▂▃▃▃▃▃▃▃▃▃▃▃▃▃▂▂▂▂▂▂▂▂ |
| AM J SURG PATHOL | 1999 | 6.37 | **2003** | 2005 | ▂▂▂▂▃▃▃▂▂▂▂▂▂▂▂▂▂▂▂▂▂▂▂▂▂ |
| EUR J CANCER PREV | 2003 | 4.88 | **2003** | 2011 | ▂▂▂▂▃▃▃▃▃▃▃▃▃▂▂▂▂▂▂▂▂▂▂▂▂ |
| INT J CANCER | 1999 | 4.79 | **2003** | 2005 | ▂▂▂▂▃▃▃▂▂▂▂▂▂▂▂▂▂▂▂▂▂▂▂▂▂ |
| PUBLIC HEALTH NUTR | 2003 | 4.46 | **2003** | 2005 | ▂▂▂▂▃▃▃▂▂▂▂▂▂▂▂▂▂▂▂▂▂▂▂▂▂ |
| J NEUROSURG | 2003 | 4.36 | **2003** | 2011 | ▂▂▂▂▃▃▃▃▃▃▃▃▃▂▂▂▂▂▂▂▂▂▂▂▂ |
| MOL CELL ENDOCRINOL | 2001 | 4.36 | **2003** | 2005 | ▂▂▂▂▃▃▃▂▂▂▂▂▂▂▂▂▂▂▂▂▂▂▂▂▂ |
| INT J ONCOL | 2003 | 4.29 | **2003** | 2007 | ▂▂▂▂▃▃▃▃▃▂▂▂▂▂▂▂▂▂▂▂▂▂▂▂▂ |
| HEPATOLOGY | 1999 | 3.92 | **2003** | 2006 | ▂▂▂▂▃▃▃▃▂▂▂▂▂▂▂▂▂▂▂▂▂▂▂▂▂ |
| CANCER DETECT PREV | 2003 | 3.29 | **2003** | 2009 | ▂▂▂▂▃▃▃▃▃▃▃▂▂▂▂▂▂▂▂▂▂▂▂▂▂ |
| TRANSPLANTATION | 2004 | 6.88 | **2004** | 2015 | ▂▂▂▂▂▃▃▃▃▃▃▃▃▃▃▃▃▂▂▂▂▂▂▂▂ |
| BIOCHEM BIOPH RES CO | 1999 | 5.78 | **2004** | 2012 | ▂▂▂▂▂▃▃▃▃▃▃▃▃▃▂▂▂▂▂▂▂▂▂▂▂ |
| CYTOPATHOLOGY | 2004 | 5.4 | **2004** | 2010 | ▂▂▂▂▂▃▃▃▃▃▃▃▂▂▂▂▂▂▂▂▂▂▂▂▂ |
| ONCOLOGY-NY | 2004 | 4.54 | **2004** | 2009 | ▂▂▂▂▂▃▃▃▃▃▃▂▂▂▂▂▂▂▂▂▂▂▂▂▂ |
| ANTICANCER RES | 1999 | 4.36 | **2004** | 2005 | ▂▂▂▂▂▃▃▂▂▂▂▂▂▂▂▂▂▂▂▂▂▂▂▂▂ |
| STEROIDS | 2004 | 4.21 | **2004** | 2012 | ▂▂▂▂▂▃▃▃▃▃▃▃▃▃▂▂▂▂▂▂▂▂▂▂▂ |
| J NUTR | 1999 | 7.88 | **2005** | 2011 | ▂▂▂▂▂▂▃▃▃▃▃▃▃▂▂▂▂▂▂▂▂▂▂▂▂ |
| CLIN CHIM ACTA | 1999 | 6.42 | **2005** | 2010 | ▂▂▂▂▂▂▃▃▃▃▃▃▂▂▂▂▂▂▂▂▂▂▂▂▂ |
| RADIAT RES | 2005 | 5.55 | **2005** | 2009 | ▂▂▂▂▂▂▃▃▃▃▃▂▂▂▂▂▂▂▂▂▂▂▂▂▂ |
| J NATL CANCER INST MONOGR | 2005 | 15.05 | **2006** | 2013 | ▂▂▂▂▂▂▂▃▃▃▃▃▃▃▃▂▂▂▂▂▂▂▂▂▂ |
| CURR OPIN OBSTET GYN | 2006 | 10.24 | **2006** | 2017 | ▂▂▂▂▂▂▂▃▃▃▃▃▃▃▃▃▃▃▃▂▂▂▂▂▂ |
| CHINESE MED J-PEKING | 2006 | 7.66 | **2006** | 2014 | ▂▂▂▂▂▂▂▃▃▃▃▃▃▃▃▃▂▂▂▂▂▂▂▂▂ |
| JNCI-J NATL CANCER I | 1999 | 5.74 | **2006** | 2007 | ▂▂▂▂▂▂▂▃▃▂▂▂▂▂▂▂▂▂▂▂▂▂▂▂▂ |
| BIOCHEM PHARMACOL | 2006 | 5.5 | **2006** | 2009 | ▂▂▂▂▂▂▂▃▃▃▃▂▂▂▂▂▂▂▂▂▂▂▂▂▂ |
| BIOCHEM J | 1999 | 5.23 | **2006** | 2011 | ▂▂▂▂▂▂▂▃▃▃▃▃▃▂▂▂▂▂▂▂▂▂▂▂▂ |
| J INFECT DIS | 2006 | 5.12 | **2006** | 2013 | ▂▂▂▂▂▂▂▃▃▃▃▃▃▃▃▂▂▂▂▂▂▂▂▂▂ |
| HAEMATOLOGICA | 2006 | 4.43 | **2006** | 2007 | ▂▂▂▂▂▂▂▃▃▂▂▂▂▂▂▂▂▂▂▂▂▂▂▂▂ |
| CLIN RADIOL | 2006 | 4.21 | **2006** | 2013 | ▂▂▂▂▂▂▂▃▃▃▃▃▃▃▃▂▂▂▂▂▂▂▂▂▂ |
| BEST PRACT RES CL EN | 2005 | 4.16 | **2006** | 2016 | ▂▂▂▂▂▂▂▃▃▃▃▃▃▃▃▃▃▃▂▂▂▂▂▂▂ |
| LEUKEMIA LYMPHOMA | 2002 | 4.13 | **2006** | 2013 | ▂▂▂▂▂▂▂▃▃▃▃▃▃▃▃▂▂▂▂▂▂▂▂▂▂ |
| J CLIN INVEST | 1999 | 3.89 | **2006** | 2008 | ▂▂▂▂▂▂▂▃▃▃▂▂▂▂▂▂▂▂▂▂▂▂▂▂▂ |
| J BIOL CHEM | 1999 | 3.57 | **2006** | 2007 | ▂▂▂▂▂▂▂▃▃▂▂▂▂▂▂▂▂▂▂▂▂▂▂▂▂ |
| HUM FERTIL (CAMB) | 2007 | 13.71 | **2007** | 2014 | ▂▂▂▂▂▂▂▂▃▃▃▃▃▃▃▃▂▂▂▂▂▂▂▂▂ |
| J AM COLL SURGEONS | 2003 | 10.22 | **2007** | 2012 | ▂▂▂▂▂▂▂▂▃▃▃▃▃▃▂▂▂▂▂▂▂▂▂▂▂ |
| ENVIRON HEALTH PERSP | 1999 | 8.84 | **2007** | 2010 | ▂▂▂▂▂▂▂▂▃▃▃▃▂▂▂▂▂▂▂▂▂▂▂▂▂ |
| NAT CLIN PRACT UROL | 2007 | 7.52 | **2007** | 2015 | ▂▂▂▂▂▂▂▂▃▃▃▃▃▃▃▃▃▂▂▂▂▂▂▂▂ |
| CURR OPIN UROL | 2007 | 7.08 | **2007** | 2016 | ▂▂▂▂▂▂▂▂▃▃▃▃▃▃▃▃▃▃▂▂▂▂▂▂▂ |
| CANCER CYTOPATHOL | 2007 | 6.22 | **2007** | 2011 | ▂▂▂▂▂▂▂▂▃▃▃▃▃▂▂▂▂▂▂▂▂▂▂▂▂ |
| J GASTROINTEST SURG | 2007 | 4.27 | **2007** | 2012 | ▂▂▂▂▂▂▂▂▃▃▃▃▃▃▂▂▂▂▂▂▂▂▂▂▂ |
| MOL PHARMACOL | 2007 | 3.59 | **2007** | 2010 | ▂▂▂▂▂▂▂▂▃▃▃▃▂▂▂▂▂▂▂▂▂▂▂▂▂ |
| J CLIN PATHOL | 1999 | 12.71 | **2008** | 2013 | ▂▂▂▂▂▂▂▂▂▃▃▃▃▃▃▂▂▂▂▂▂▂▂▂▂ |
| THYROID | 2008 | 8.13 | **2008** | 2015 | ▂▂▂▂▂▂▂▂▂▃▃▃▃▃▃▃▃▂▂▂▂▂▂▂▂ |
| APMIS | 1999 | 7.54 | **2008** | 2011 | ▂▂▂▂▂▂▂▂▂▃▃▃▃▂▂▂▂▂▂▂▂▂▂▂▂ |
| JPN J CLIN ONCOL | 2008 | 4.61 | **2008** | 2011 | ▂▂▂▂▂▂▂▂▂▃▃▃▃▂▂▂▂▂▂▂▂▂▂▂▂ |
| J ROY SOC MED | 2008 | 4.38 | **2008** | 2012 | ▂▂▂▂▂▂▂▂▂▃▃▃▃▃▂▂▂▂▂▂▂▂▂▂▂ |
| EUR J OBSTET GYN R B | 2005 | 4.07 | **2008** | 2010 | ▂▂▂▂▂▂▂▂▂▃▃▃▂▂▂▂▂▂▂▂▂▂▂▂▂ |
| J PEDIAT HEMATOL ONC | 2000 | 4.02 | **2008** | 2010 | ▂▂▂▂▂▂▂▂▂▃▃▃▂▂▂▂▂▂▂▂▂▂▂▂▂ |
| REPROD TOXICOL | 2004 | 3.85 | **2008** | 2009 | ▂▂▂▂▂▂▂▂▂▃▃▂▂▂▂▂▂▂▂▂▂▂▂▂▂ |
| WHO LABORATORY MANUAL FOR THE EXAMINATION OF HUMAN SEMEN AND SPERM-CERVICAL MUCUS INTERACTION | 2009 | 10.49 | **2009** | 2013 | ▂▂▂▂▂▂▂▂▂▂▃▃▃▃▃▂▂▂▂▂▂▂▂▂▂ |
| GYNECOL ONCOL | 2000 | 8.03 | **2009** | 2014 | ▂▂▂▂▂▂▂▂▂▂▃▃▃▃▃▃▂▂▂▂▂▂▂▂▂ |
| ANN DIAGN PATHOL | 2009 | 6.3 | **2009** | 2014 | ▂▂▂▂▂▂▂▂▂▂▃▃▃▃▃▃▂▂▂▂▂▂▂▂▂ |
| LANGENBECK ARCH SURG | 2009 | 5.65 | **2009** | 2012 | ▂▂▂▂▂▂▂▂▂▂▃▃▃▃▂▂▂▂▂▂▂▂▂▂▂ |
| CANCER EPIDEM BIOMAR | 2000 | 4.93 | **2009** | 2012 | ▂▂▂▂▂▂▂▂▂▂▃▃▃▃▂▂▂▂▂▂▂▂▂▂▂ |
| BRIT J PHARMACOL | 2009 | 4.46 | **2009** | 2010 | ▂▂▂▂▂▂▂▂▂▂▃▃▂▂▂▂▂▂▂▂▂▂▂▂▂ |
| AM J CLIN ONCOL-CANC | 2004 | 4.05 | **2009** | 2014 | ▂▂▂▂▂▂▂▂▂▂▃▃▃▃▃▃▂▂▂▂▂▂▂▂▂ |
| TRANSFUSION | 2009 | 3.96 | **2009** | 2018 | ▂▂▂▂▂▂▂▂▂▂▃▃▃▃▃▃▃▃▃▃▂▂▂▂▂ |
| EUR J NUCL MED MOL I | 2009 | 3.82 | **2009** | 2010 | ▂▂▂▂▂▂▂▂▂▂▃▃▂▂▂▂▂▂▂▂▂▂▂▂▂ |
| ANZ J SURG | 2005 | 3.73 | **2009** | 2012 | ▂▂▂▂▂▂▂▂▂▂▃▃▃▃▂▂▂▂▂▂▂▂▂▂▂ |
| J CLIN MICROBIOL | 2009 | 3.67 | **2009** | 2013 | ▂▂▂▂▂▂▂▂▂▂▃▃▃▃▃▂▂▂▂▂▂▂▂▂▂ |
| LUNG CANCER | 2010 | 6.64 | **2010** | 2013 | ▂▂▂▂▂▂▂▂▂▂▂▃▃▃▃▂▂▂▂▂▂▂▂▂▂ |
| ONCOLOGIST | 2007 | 6.05 | **2010** | 2015 | ▂▂▂▂▂▂▂▂▂▂▂▃▃▃▃▃▃▂▂▂▂▂▂▂▂ |
| ENDOCR-RELAT CANCER | 2007 | 5.68 | **2010** | 2017 | ▂▂▂▂▂▂▂▂▂▂▂▃▃▃▃▃▃▃▃▂▂▂▂▂▂ |
| SEMIN ONCOL | 1999 | 5.07 | **2010** | 2012 | ▂▂▂▂▂▂▂▂▂▂▂▃▃▃▂▂▂▂▂▂▂▂▂▂▂ |
| V138 | 2010 | 4.65 | **2010** | 2018 | ▂▂▂▂▂▂▂▂▂▂▂▃▃▃▃▃▃▃▃▃▂▂▂▂▂ |
| J IMMUNOL | 2000 | 4.17 | **2010** | 2012 | ▂▂▂▂▂▂▂▂▂▂▂▃▃▃▂▂▂▂▂▂▂▂▂▂▂ |
| ANN NY ACAD SCI | 1999 | 3.82 | **2010** | 2014 | ▂▂▂▂▂▂▂▂▂▂▂▃▃▃▃▃▂▂▂▂▂▂▂▂▂ |
| INT J OBESITY | 2010 | 3.76 | **2010** | 2015 | ▂▂▂▂▂▂▂▂▂▂▂▃▃▃▃▃▃▂▂▂▂▂▂▂▂ |
| NEUROUROL URODYNAM | 2011 | 7.1 | **2011** | 2016 | ▂▂▂▂▂▂▂▂▂▂▂▂▃▃▃▃▃▃▂▂▂▂▂▂▂ |
| STEM CELLS | 2011 | 6.58 | **2011** | 2014 | ▂▂▂▂▂▂▂▂▂▂▂▂▃▃▃▃▂▂▂▂▂▂▂▂▂ |
| J MED FOOD | 2011 | 3.98 | **2011** | 2015 | ▂▂▂▂▂▂▂▂▂▂▂▂▃▃▃▃▃▂▂▂▂▂▂▂▂ |
| EFF CANC TREATM REPR | 2011 | 3.79 | **2011** | 2014 | ▂▂▂▂▂▂▂▂▂▂▂▂▃▃▃▃▂▂▂▂▂▂▂▂▂ |
| MAYO CLIN PROC | 2003 | 3.68 | **2011** | 2012 | ▂▂▂▂▂▂▂▂▂▂▂▂▃▃▂▂▂▂▂▂▂▂▂▂▂ |
| HISTOL HISTOPATHOL | 2011 | 3.55 | **2011** | 2016 | ▂▂▂▂▂▂▂▂▂▂▂▂▃▃▃▃▃▃▂▂▂▂▂▂▂ |
| FOOD CHEM | 2011 | 3.48 | **2011** | 2015 | ▂▂▂▂▂▂▂▂▂▂▂▂▃▃▃▃▃▂▂▂▂▂▂▂▂ |
| INT J DEV BIOL | 2011 | 3.48 | **2011** | 2015 | ▂▂▂▂▂▂▂▂▂▂▂▂▃▃▃▃▃▂▂▂▂▂▂▂▂ |
| SEMIN REPROD MED | 2012 | 12.59 | **2012** | 2018 | ▂▂▂▂▂▂▂▂▂▂▂▂▂▃▃▃▃▃▃▃▂▂▂▂▂ |
| CLIN OBSTET GYNECOL | 2012 | 9.44 | **2012** | 2016 | ▂▂▂▂▂▂▂▂▂▂▂▂▂▃▃▃▃▃▂▂▂▂▂▂▂ |
| CLIN J ONCOL NURS | 2012 | 6.42 | **2012** | 2019 | ▂▂▂▂▂▂▂▂▂▂▂▂▂▃▃▃▃▃▃▃▃▂▂▂▂ |
| PATHOL INT | 2000 | 5.57 | **2012** | 2016 | ▂▂▂▂▂▂▂▂▂▂▂▂▂▃▃▃▃▃▂▂▂▂▂▂▂ |
| CLIN ORTHOP RELAT R | 2012 | 5.54 | **2012** | 2016 | ▂▂▂▂▂▂▂▂▂▂▂▂▂▃▃▃▃▃▂▂▂▂▂▂▂ |
| VIRCHOWS ARCH | 2004 | 4.12 | **2012** | 2014 | ▂▂▂▂▂▂▂▂▂▂▂▂▂▃▃▃▂▂▂▂▂▂▂▂▂ |
| CANCER J | 2012 | 3.97 | **2012** | 2013 | ▂▂▂▂▂▂▂▂▂▂▂▂▂▃▃▂▂▂▂▂▂▂▂▂▂ |
| APPL IMMUNOHISTO M M | 2004 | 3.92 | **2012** | 2015 | ▂▂▂▂▂▂▂▂▂▂▂▂▂▃▃▃▃▂▂▂▂▂▂▂▂ |
| PSYCHO-ONCOL | 2009 | 9.54 | **2013** | 2018 | ▂▂▂▂▂▂▂▂▂▂▂▂▂▂▃▃▃▃▃▃▂▂▂▂▂ |
| BJOG-INT J OBSTET GY | 2010 | 8.88 | **2013** | 2020 | ▂▂▂▂▂▂▂▂▂▂▂▂▂▂▃▃▃▃▃▃▃▃▂▂▂ |
| PROSTATE CANCER P D | 2013 | 7.87 | **2013** | 2016 | ▂▂▂▂▂▂▂▂▂▂▂▂▂▂▃▃▃▃▂▂▂▂▂▂▂ |
| CURR TREAT OPTION ON | 2013 | 7.25 | **2013** | 2019 | ▂▂▂▂▂▂▂▂▂▂▂▂▂▂▃▃▃▃▃▃▃▂▂▂▂ |
| HUM FERTIL | 2011 | 5.21 | **2013** | 2019 | ▂▂▂▂▂▂▂▂▂▂▂▂▂▂▃▃▃▃▃▃▃▂▂▂▂ |
| INT BRAZ J UROL | 2013 | 5.17 | **2013** | 2018 | ▂▂▂▂▂▂▂▂▂▂▂▂▂▂▃▃▃▃▃▃▂▂▂▂▂ |
| J CLIN EPIDEMIOL | 1999 | 5.16 | **2013** | 2015 | ▂▂▂▂▂▂▂▂▂▂▂▂▂▂▃▃▃▂▂▂▂▂▂▂▂ |
| UROL INT | 2003 | 5.01 | **2013** | 2015 | ▂▂▂▂▂▂▂▂▂▂▂▂▂▂▃▃▃▂▂▂▂▂▂▂▂ |
| WORLD J UROL | 2006 | 4.53 | **2013** | 2015 | ▂▂▂▂▂▂▂▂▂▂▂▂▂▂▃▃▃▂▂▂▂▂▂▂▂ |
| MATURITAS | 2013 | 4.45 | **2013** | 2015 | ▂▂▂▂▂▂▂▂▂▂▂▂▂▂▃▃▃▂▂▂▂▂▂▂▂ |
| CYTOKINE | 2013 | 3.76 | **2013** | 2016 | ▂▂▂▂▂▂▂▂▂▂▂▂▂▂▃▃▃▃▂▂▂▂▂▂▂ |
| HEALTH QUAL LIFE OUT | 2013 | 3.7 | **2013** | 2018 | ▂▂▂▂▂▂▂▂▂▂▂▂▂▂▃▃▃▃▃▃▂▂▂▂▂ |
| ARCH PHYS MED REHAB | 2013 | 3.68 | **2013** | 2014 | ▂▂▂▂▂▂▂▂▂▂▂▂▂▂▃▃▂▂▂▂▂▂▂▂▂ |
| QUAL LIFE RES | 2013 | 3.58 | **2013** | 2016 | ▂▂▂▂▂▂▂▂▂▂▂▂▂▂▃▃▃▃▂▂▂▂▂▂▂ |
| NAT REV GENET | 2013 | 3.29 | **2013** | 2018 | ▂▂▂▂▂▂▂▂▂▂▂▂▂▂▃▃▃▃▃▃▂▂▂▂▂ |
| HAEMATOL-HEMATOL J | 2014 | 6.62 | **2014** | 2018 | ▂▂▂▂▂▂▂▂▂▂▂▂▂▂▂▃▃▃▃▃▂▂▂▂▂ |
| CANCER TREAT RES | 2014 | 6.29 | **2014** | 2019 | ▂▂▂▂▂▂▂▂▂▂▂▂▂▂▂▃▃▃▃▃▃▂▂▂▂ |
| CANCER TREAT REV | 2005 | 5.67 | **2014** | 2017 | ▂▂▂▂▂▂▂▂▂▂▂▂▂▂▂▃▃▃▃▂▂▂▂▂▂ |
| ACTA PAEDIATR | 2001 | 5.32 | **2014** | 2019 | ▂▂▂▂▂▂▂▂▂▂▂▂▂▂▂▃▃▃▃▃▃▂▂▂▂ |
| MED CARE | 2014 | 4.97 | **2014** | 2018 | ▂▂▂▂▂▂▂▂▂▂▂▂▂▂▂▃▃▃▃▃▂▂▂▂▂ |
| INT J UROL | 2003 | 4.87 | **2014** | 2015 | ▂▂▂▂▂▂▂▂▂▂▂▂▂▂▂▃▃▂▂▂▂▂▂▂▂ |
| EUR J CARDIO-THORAC | 2014 | 4.74 | **2014** | 2016 | ▂▂▂▂▂▂▂▂▂▂▂▂▂▂▂▃▃▃▂▂▂▂▂▂▂ |
| CELL STEM CELL | 2013 | 4.59 | **2014** | 2020 | ▂▂▂▂▂▂▂▂▂▂▂▂▂▂▂▃▃▃▃▃▃▃▂▂▂ |
| J BONE JOINT SURG AM | 2014 | 4.42 | **2014** | 2018 | ▂▂▂▂▂▂▂▂▂▂▂▂▂▂▂▃▃▃▃▃▂▂▂▂▂ |
| J CANCER RES CLIN | 2003 | 4.25 | **2014** | 2016 | ▂▂▂▂▂▂▂▂▂▂▂▂▂▂▂▃▃▃▂▂▂▂▂▂▂ |
| WHO CLASSIFICATION T | 2014 | 4.07 | **2014** | 2019 | ▂▂▂▂▂▂▂▂▂▂▂▂▂▂▂▃▃▃▃▃▃▂▂▂▂ |
| MOL HUM REPROD | 2002 | 3.72 | **2014** | 2020 | ▂▂▂▂▂▂▂▂▂▂▂▂▂▂▂▃▃▃▃▃▃▃▂▂▂ |
| ARCH BIOCHEM BIOPHYS | 1999 | 3.53 | **2014** | 2017 | ▂▂▂▂▂▂▂▂▂▂▂▂▂▂▂▃▃▃▃▂▂▂▂▂▂ |
| EUR J HUM GENET | 2014 | 3.33 | **2014** | 2019 | ▂▂▂▂▂▂▂▂▂▂▂▂▂▂▂▃▃▃▃▃▃▂▂▂▂ |
| ANAL CHEM | 2014 | 3.32 | **2014** | 2015 | ▂▂▂▂▂▂▂▂▂▂▂▂▂▂▂▃▃▂▂▂▂▂▂▂▂ |
| NAT REV ENDOCRINOL | 2015 | 12.09 | **2015** | 2020 | ▂▂▂▂▂▂▂▂▂▂▂▂▂▂▂▂▃▃▃▃▃▃▂▂▂ |
| J NATL COMPR CANC NE | 2015 | 10.7 | **2015** | 2018 | ▂▂▂▂▂▂▂▂▂▂▂▂▂▂▂▂▃▃▃▃▂▂▂▂▂ |
| TOXICOL APPL PHARM | 2006 | 7.7 | **2015** | 2017 | ▂▂▂▂▂▂▂▂▂▂▂▂▂▂▂▂▃▃▃▂▂▂▂▂▂ |
| PATIENT EDUC COUNS | 2011 | 7.53 | **2015** | 2020 | ▂▂▂▂▂▂▂▂▂▂▂▂▂▂▂▂▃▃▃▃▃▃▂▂▂ |
| GYNECOL ENDOCRINOL | 2015 | 7.51 | **2015** | 2021 | ▂▂▂▂▂▂▂▂▂▂▂▂▂▂▂▂▃▃▃▃▃▃▃▂▂ |
| J SURG RES | 2002 | 6.28 | **2015** | 2018 | ▂▂▂▂▂▂▂▂▂▂▂▂▂▂▂▂▃▃▃▃▂▂▂▂▂ |
| BIOMED RES INT | 2015 | 5.93 | **2015** | 2023 | ▂▂▂▂▂▂▂▂▂▂▂▂▂▂▂▂▃▃▃▃▃▃▃▃▃ |
| ANIM REPROD SCI | 2004 | 5.79 | **2015** | 2023 | ▂▂▂▂▂▂▂▂▂▂▂▂▂▂▂▂▃▃▃▃▃▃▃▃▃ |
| TRENDS ENDOCRIN MET | 2015 | 5.66 | **2015** | 2021 | ▂▂▂▂▂▂▂▂▂▂▂▂▂▂▂▂▃▃▃▃▃▃▃▂▂ |
| METABOLISM | 2010 | 5.3 | **2015** | 2020 | ▂▂▂▂▂▂▂▂▂▂▂▂▂▂▂▂▃▃▃▃▃▃▂▂▂ |
| THERIOGENOLOGY | 2008 | 4.94 | **2015** | 2017 | ▂▂▂▂▂▂▂▂▂▂▂▂▂▂▂▂▃▃▃▂▂▂▂▂▂ |
| ACTA NEUROPATHOL | 1999 | 4.5 | **2015** | 2016 | ▂▂▂▂▂▂▂▂▂▂▂▂▂▂▂▂▃▃▂▂▂▂▂▂▂ |
| LIFE SCI | 2006 | 4.25 | **2015** | 2019 | ▂▂▂▂▂▂▂▂▂▂▂▂▂▂▂▂▃▃▃▃▃▂▂▂▂ |
| ATHEROSCLEROSIS | 2015 | 4.1 | **2015** | 2019 | ▂▂▂▂▂▂▂▂▂▂▂▂▂▂▂▂▃▃▃▃▃▂▂▂▂ |
| BEST PRACT RES CL OB | 2012 | 3.58 | **2015** | 2017 | ▂▂▂▂▂▂▂▂▂▂▂▂▂▂▂▂▃▃▃▂▂▂▂▂▂ |
| J WOMENS HEALTH | 2016 | 7.72 | **2016** | 2019 | ▂▂▂▂▂▂▂▂▂▂▂▂▂▂▂▂▂▃▃▃▃▂▂▂▂ |
| J SEX MED | 2013 | 7.61 | **2016** | 2019 | ▂▂▂▂▂▂▂▂▂▂▂▂▂▂▂▂▂▃▃▃▃▂▂▂▂ |
| LANCET DIABETES ENDO | 2016 | 7.34 | **2016** | 2023 | ▂▂▂▂▂▂▂▂▂▂▂▂▂▂▂▂▂▃▃▃▃▃▃▃▃ |
| CURR ONCOL | 2016 | 7.17 | **2016** | 2019 | ▂▂▂▂▂▂▂▂▂▂▂▂▂▂▂▂▂▃▃▃▃▂▂▂▂ |
| SOC SCI MED | 2011 | 6.33 | **2016** | 2017 | ▂▂▂▂▂▂▂▂▂▂▂▂▂▂▂▂▂▃▃▂▂▂▂▂▂ |
| J PEDIATR ONCOL NURS | 2009 | 6.11 | **2016** | 2019 | ▂▂▂▂▂▂▂▂▂▂▂▂▂▂▂▂▂▃▃▃▃▂▂▂▂ |
| MOL NUTR FOOD RES | 2016 | 5.85 | **2016** | 2017 | ▂▂▂▂▂▂▂▂▂▂▂▂▂▂▂▂▂▃▃▂▂▂▂▂▂ |
| GENOME RES | 2016 | 4.82 | **2016** | 2018 | ▂▂▂▂▂▂▂▂▂▂▂▂▂▂▂▂▂▃▃▃▂▂▂▂▂ |
| J PSYCHOSOM OBST GYN | 2016 | 4.68 | **2016** | 2017 | ▂▂▂▂▂▂▂▂▂▂▂▂▂▂▂▂▂▃▃▂▂▂▂▂▂ |
| PLOS GENET | 2012 | 4.67 | **2016** | 2017 | ▂▂▂▂▂▂▂▂▂▂▂▂▂▂▂▂▂▃▃▂▂▂▂▂▂ |
| BIOMATERIALS | 2008 | 4.34 | **2016** | 2019 | ▂▂▂▂▂▂▂▂▂▂▂▂▂▂▂▂▂▃▃▃▃▂▂▂▂ |
| INT J SURG CASE REP | 2016 | 3.79 | **2016** | 2020 | ▂▂▂▂▂▂▂▂▂▂▂▂▂▂▂▂▂▃▃▃▃▃▂▂▂ |
| HUM MOL GENET | 2000 | 3.67 | **2016** | 2018 | ▂▂▂▂▂▂▂▂▂▂▂▂▂▂▂▂▂▃▃▃▂▂▂▂▂ |
| BMC CANCER | 2011 | 12.33 | **2017** | 2023 | ▂▂▂▂▂▂▂▂▂▂▂▂▂▂▂▂▂▂▃▃▃▃▃▃▃ |
| EUR J CANCER CARE | 2012 | 12.24 | **2017** | 2021 | ▂▂▂▂▂▂▂▂▂▂▂▂▂▂▂▂▂▂▃▃▃▃▃▂▂ |
| J ADOLESCENT HEALTH | 2009 | 9.78 | **2017** | 2020 | ▂▂▂▂▂▂▂▂▂▂▂▂▂▂▂▂▂▂▃▃▃▃▂▂▂ |
| MOL CELL BIOL | 2012 | 9.63 | **2017** | 2021 | ▂▂▂▂▂▂▂▂▂▂▂▂▂▂▂▂▂▂▃▃▃▃▃▂▂ |
| REPROD BIOL ENDOCRIN | 2014 | 9.54 | **2017** | 2023 | ▂▂▂▂▂▂▂▂▂▂▂▂▂▂▂▂▂▂▃▃▃▃▃▃▃ |
| J PSYCHOSOC ONCOL | 2016 | 9.45 | **2017** | 2021 | ▂▂▂▂▂▂▂▂▂▂▂▂▂▂▂▂▂▂▃▃▃▃▃▂▂ |
| LEUKEMIA | 2015 | 9.39 | **2017** | 2021 | ▂▂▂▂▂▂▂▂▂▂▂▂▂▂▂▂▂▂▃▃▃▃▃▂▂ |
| SCI TRANSL MED | 2015 | 7.6 | **2017** | 2021 | ▂▂▂▂▂▂▂▂▂▂▂▂▂▂▂▂▂▂▃▃▃▃▃▂▂ |
| J CANCER EDUC | 2017 | 7.2 | **2017** | 2021 | ▂▂▂▂▂▂▂▂▂▂▂▂▂▂▂▂▂▂▃▃▃▃▃▂▂ |
| FUTURE ONCOL | 2017 | 6.98 | **2017** | 2020 | ▂▂▂▂▂▂▂▂▂▂▂▂▂▂▂▂▂▂▃▃▃▃▂▂▂ |
| CLIN GENITOURIN CANC | 2017 | 6.81 | **2017** | 2019 | ▂▂▂▂▂▂▂▂▂▂▂▂▂▂▂▂▂▂▃▃▃▂▂▂▂ |
| HORM RES | 2004 | 6.03 | **2017** | 2020 | ▂▂▂▂▂▂▂▂▂▂▂▂▂▂▂▂▂▂▃▃▃▃▂▂▂ |
| MOL ENDOCRINOL | 2017 | 5.62 | **2017** | 2020 | ▂▂▂▂▂▂▂▂▂▂▂▂▂▂▂▂▂▂▃▃▃▃▂▂▂ |
| BRAIN BEHAV IMMUN | 2017 | 4.76 | **2017** | 2020 | ▂▂▂▂▂▂▂▂▂▂▂▂▂▂▂▂▂▂▃▃▃▃▂▂▂ |
| J THORAC ONCOL | 2017 | 4.71 | **2017** | 2018 | ▂▂▂▂▂▂▂▂▂▂▂▂▂▂▂▂▂▂▃▃▂▂▂▂▂ |
| J REPROD FERTIL | 2000 | 4.41 | **2017** | 2018 | ▂▂▂▂▂▂▂▂▂▂▂▂▂▂▂▂▂▂▃▃▂▂▂▂▂ |
| TISSUE CELL | 2002 | 4.41 | **2017** | 2020 | ▂▂▂▂▂▂▂▂▂▂▂▂▂▂▂▂▂▂▃▃▃▃▂▂▂ |
| HORM-INT J ENDOCRINO | 2017 | 4.29 | **2017** | 2023 | ▂▂▂▂▂▂▂▂▂▂▂▂▂▂▂▂▂▂▃▃▃▃▃▃▃ |
| TOXICOL SCI | 2017 | 4.16 | **2017** | 2023 | ▂▂▂▂▂▂▂▂▂▂▂▂▂▂▂▂▂▂▃▃▃▃▃▃▃ |
| EUR J PEDIATR | 2008 | 4.07 | **2017** | 2021 | ▂▂▂▂▂▂▂▂▂▂▂▂▂▂▂▂▂▂▃▃▃▃▃▂▂ |
| J PEDIATR-US | 2001 | 3.77 | **2017** | 2021 | ▂▂▂▂▂▂▂▂▂▂▂▂▂▂▂▂▂▂▃▃▃▃▃▂▂ |
| NAT PROTOC | 2011 | 3.77 | **2017** | 2019 | ▂▂▂▂▂▂▂▂▂▂▂▂▂▂▂▂▂▂▃▃▃▂▂▂▂ |
| PEDIATR HEMAT ONCOL | 2011 | 3.75 | **2017** | 2018 | ▂▂▂▂▂▂▂▂▂▂▂▂▂▂▂▂▂▂▃▃▂▂▂▂▂ |
| MICROSC RES TECHNIQ | 2017 | 3.54 | **2017** | 2021 | ▂▂▂▂▂▂▂▂▂▂▂▂▂▂▂▂▂▂▃▃▃▃▃▂▂ |
| J CELL BIOCHEM | 2009 | 3.39 | **2017** | 2021 | ▂▂▂▂▂▂▂▂▂▂▂▂▂▂▂▂▂▂▃▃▃▃▃▂▂ |
| CANCER PREV RES | 2011 | 3.28 | **2017** | 2021 | ▂▂▂▂▂▂▂▂▂▂▂▂▂▂▂▂▂▂▃▃▃▃▃▂▂ |
| SUPPORT CARE CANCER | 2013 | 21.58 | **2018** | 2023 | ▂▂▂▂▂▂▂▂▂▂▂▂▂▂▂▂▂▂▂▃▃▃▃▃▃ |
| J ADOLESC YOUNG ADUL | 2016 | 21.52 | **2018** | 2023 | ▂▂▂▂▂▂▂▂▂▂▂▂▂▂▂▂▂▂▂▃▃▃▃▃▃ |
| ANDROLOGY-US | 2014 | 21 | **2018** | 2023 | ▂▂▂▂▂▂▂▂▂▂▂▂▂▂▂▂▂▂▂▃▃▃▃▃▃ |
| NAT COMMUN | 2016 | 20.99 | **2018** | 2023 | ▂▂▂▂▂▂▂▂▂▂▂▂▂▂▂▂▂▂▂▃▃▃▃▃▃ |
| UROL ONCOL-SEMIN ORI | 2013 | 16.15 | **2018** | 2023 | ▂▂▂▂▂▂▂▂▂▂▂▂▂▂▂▂▂▂▂▃▃▃▃▃▃ |
| J ONCOL PRACT | 2016 | 8.25 | **2018** | 2023 | ▂▂▂▂▂▂▂▂▂▂▂▂▂▂▂▂▂▂▂▃▃▃▃▃▃ |
| WHO LABORATORY MANUAL FOR THE EXAMINATION AND PROCESSING OF HUMAN SEMEN | 2016 | 7.64 | **2018** | 2021 | ▂▂▂▂▂▂▂▂▂▂▂▂▂▂▂▂▂▂▂▃▃▃▃▂▂ |
| CURR OPIN ENDOCRINOL | 2016 | 7.31 | **2018** | 2020 | ▂▂▂▂▂▂▂▂▂▂▂▂▂▂▂▂▂▂▂▃▃▃▂▂▂ |
| ARCH SEX BEHAV | 2018 | 7.31 | **2018** | 2020 | ▂▂▂▂▂▂▂▂▂▂▂▂▂▂▂▂▂▂▂▃▃▃▂▂▂ |
| NAT REV UROL | 2014 | 7.17 | **2018** | 2023 | ▂▂▂▂▂▂▂▂▂▂▂▂▂▂▂▂▂▂▂▃▃▃▃▃▃ |
| WORLD J SURG ONCOL | 2015 | 6.97 | **2018** | 2020 | ▂▂▂▂▂▂▂▂▂▂▂▂▂▂▂▂▂▂▂▃▃▃▂▂▂ |
| EUR J PHARMACOL | 2018 | 6.33 | **2018** | 2020 | ▂▂▂▂▂▂▂▂▂▂▂▂▂▂▂▂▂▂▂▃▃▃▂▂▂ |
| RADIOGRAPHICS | 2012 | 6.23 | **2018** | 2021 | ▂▂▂▂▂▂▂▂▂▂▂▂▂▂▂▂▂▂▂▃▃▃▃▂▂ |
| MOL CLIN ONCOL | 2018 | 6.14 | **2018** | 2021 | ▂▂▂▂▂▂▂▂▂▂▂▂▂▂▂▂▂▂▂▃▃▃▃▂▂ |
| J PEDIATR ENDOCR MET | 2018 | 6.09 | **2018** | 2019 | ▂▂▂▂▂▂▂▂▂▂▂▂▂▂▂▂▂▂▂▃▃▂▂▂▂ |
| METHODS MOL BIOL | 2014 | 5.87 | **2018** | 2023 | ▂▂▂▂▂▂▂▂▂▂▂▂▂▂▂▂▂▂▂▃▃▃▃▃▃ |
| EUR J ONCOL NURS | 2010 | 5.77 | **2018** | 2019 | ▂▂▂▂▂▂▂▂▂▂▂▂▂▂▂▂▂▂▂▃▃▂▂▂▂ |
| AM J PHYSIOL-ENDOC M | 2008 | 4.83 | **2018** | 2020 | ▂▂▂▂▂▂▂▂▂▂▂▂▂▂▂▂▂▂▂▃▃▃▂▂▂ |
| PSYCHO-ONCOLOGY | 2009 | 4.78 | **2018** | 2021 | ▂▂▂▂▂▂▂▂▂▂▂▂▂▂▂▂▂▂▂▃▃▃▃▂▂ |
| EUR J HAEMATOL | 2018 | 4.5 | **2018** | 2021 | ▂▂▂▂▂▂▂▂▂▂▂▂▂▂▂▂▂▂▂▃▃▃▃▂▂ |
| J ANAT | 2018 | 4.33 | **2018** | 2020 | ▂▂▂▂▂▂▂▂▂▂▂▂▂▂▂▂▂▂▂▃▃▃▂▂▂ |
| ANN RHEUM DIS | 2018 | 4.09 | **2018** | 2023 | ▂▂▂▂▂▂▂▂▂▂▂▂▂▂▂▂▂▂▂▃▃▃▃▃▃ |
| CLIN GASTROENTEROL H | 2018 | 3.68 | **2018** | 2021 | ▂▂▂▂▂▂▂▂▂▂▂▂▂▂▂▂▂▂▂▃▃▃▃▂▂ |
| INT J TRANSGENDERISM | 2018 | 3.68 | **2018** | 2021 | ▂▂▂▂▂▂▂▂▂▂▂▂▂▂▂▂▂▂▂▃▃▃▃▂▂ |
| PEDIATRICS | 2000 | 3.6 | **2018** | 2019 | ▂▂▂▂▂▂▂▂▂▂▂▂▂▂▂▂▂▂▂▃▃▂▂▂▂ |
| BIOL BLOOD MARROW TR | 2013 | 3.58 | **2018** | 2023 | ▂▂▂▂▂▂▂▂▂▂▂▂▂▂▂▂▂▂▂▃▃▃▃▃▃ |
| SCI REP-UK | 2017 | 41.44 | **2019** | 2023 | ▂▂▂▂▂▂▂▂▂▂▂▂▂▂▂▂▂▂▂▂▃▃▃▃▃ |
| ONCOTARGET | 2016 | 18.81 | **2019** | 2023 | ▂▂▂▂▂▂▂▂▂▂▂▂▂▂▂▂▂▂▂▂▃▃▃▃▃ |
| MEDICINE | 2001 | 18.41 | **2019** | 2023 | ▂▂▂▂▂▂▂▂▂▂▂▂▂▂▂▂▂▂▂▂▃▃▃▃▃ |
| INT J CLIN ONCOL | 2016 | 14.14 | **2019** | 2023 | ▂▂▂▂▂▂▂▂▂▂▂▂▂▂▂▂▂▂▂▂▃▃▃▃▃ |
| BMC MED | 2017 | 13.44 | **2019** | 2023 | ▂▂▂▂▂▂▂▂▂▂▂▂▂▂▂▂▂▂▂▂▃▃▃▃▃ |
| WORLD J MENS HEALTH | 2019 | 12.83 | **2019** | 2023 | ▂▂▂▂▂▂▂▂▂▂▂▂▂▂▂▂▂▂▂▂▃▃▃▃▃ |
| BASIC CLIN ANDROL | 2019 | 12.54 | **2019** | 2023 | ▂▂▂▂▂▂▂▂▂▂▂▂▂▂▂▂▂▂▂▂▃▃▃▃▃ |
| REPROD MED BIOL | 2019 | 11.95 | **2019** | 2023 | ▂▂▂▂▂▂▂▂▂▂▂▂▂▂▂▂▂▂▂▂▃▃▃▃▃ |
| J PEDIATR UROL | 2019 | 10.2 | **2019** | 2023 | ▂▂▂▂▂▂▂▂▂▂▂▂▂▂▂▂▂▂▂▂▃▃▃▃▃ |
| FRONT PHYSIOL | 2019 | 10.2 | **2019** | 2023 | ▂▂▂▂▂▂▂▂▂▂▂▂▂▂▂▂▂▂▂▂▃▃▃▃▃ |
| NUTRIENTS | 2019 | 9.91 | **2019** | 2023 | ▂▂▂▂▂▂▂▂▂▂▂▂▂▂▂▂▂▂▂▂▃▃▃▃▃ |
| CELL RES | 2017 | 8.3 | **2019** | 2023 | ▂▂▂▂▂▂▂▂▂▂▂▂▂▂▂▂▂▂▂▂▃▃▃▃▃ |
| ASIAN PAC J CANCER PREV | 2019 | 7.66 | **2019** | 2023 | ▂▂▂▂▂▂▂▂▂▂▂▂▂▂▂▂▂▂▂▂▃▃▃▃▃ |
| ENDOCR CONNECT | 2019 | 7.66 | **2019** | 2020 | ▂▂▂▂▂▂▂▂▂▂▂▂▂▂▂▂▂▂▂▂▃▃▂▂▂ |
| CURR UROL REP | 2007 | 6.94 | **2019** | 2023 | ▂▂▂▂▂▂▂▂▂▂▂▂▂▂▂▂▂▂▂▂▃▃▃▃▃ |
| REPROD HEALTH | 2019 | 6.88 | **2019** | 2021 | ▂▂▂▂▂▂▂▂▂▂▂▂▂▂▂▂▂▂▂▂▃▃▃▂▂ |
| CELL DEATH DIFFER | 2019 | 6.56 | **2019** | 2023 | ▂▂▂▂▂▂▂▂▂▂▂▂▂▂▂▂▂▂▂▂▃▃▃▃▃ |
| ELIFE | 2019 | 6.56 | **2019** | 2023 | ▂▂▂▂▂▂▂▂▂▂▂▂▂▂▂▂▂▂▂▂▃▃▃▃▃ |
| BMJ OPEN | 2019 | 6.2 | **2019** | 2023 | ▂▂▂▂▂▂▂▂▂▂▂▂▂▂▂▂▂▂▂▂▃▃▃▃▃ |
| INT J NURS STUD | 2019 | 5.91 | **2019** | 2020 | ▂▂▂▂▂▂▂▂▂▂▂▂▂▂▂▂▂▂▂▂▃▃▂▂▂ |
| ARCH GYNECOL OBSTET | 2013 | 5.83 | **2019** | 2023 | ▂▂▂▂▂▂▂▂▂▂▂▂▂▂▂▂▂▂▂▂▃▃▃▃▃ |
| GENOME BIOL | 2012 | 5.41 | **2019** | 2023 | ▂▂▂▂▂▂▂▂▂▂▂▂▂▂▂▂▂▂▂▂▃▃▃▃▃ |
| PHYSIOL REV | 2001 | 5.27 | **2019** | 2020 | ▂▂▂▂▂▂▂▂▂▂▂▂▂▂▂▂▂▂▂▂▃▃▂▂▂ |
| EUR RADIOL | 2013 | 5.13 | **2019** | 2023 | ▂▂▂▂▂▂▂▂▂▂▂▂▂▂▂▂▂▂▂▂▃▃▃▃▃ |
| THER ADV MED ONCOL | 2019 | 5.05 | **2019** | 2021 | ▂▂▂▂▂▂▂▂▂▂▂▂▂▂▂▂▂▂▂▂▃▃▃▂▂ |
| ENDOCRINE | 2014 | 4.74 | **2019** | 2020 | ▂▂▂▂▂▂▂▂▂▂▂▂▂▂▂▂▂▂▂▂▃▃▂▂▂ |
| BMC UROL | 2019 | 4.37 | **2019** | 2023 | ▂▂▂▂▂▂▂▂▂▂▂▂▂▂▂▂▂▂▂▂▃▃▃▃▃ |
| J PHYSIOL-LONDON | 2019 | 4.01 | **2019** | 2023 | ▂▂▂▂▂▂▂▂▂▂▂▂▂▂▂▂▂▂▂▂▃▃▃▃▃ |
| OTOLARYNG HEAD NECK | 2005 | 3.89 | **2019** | 2021 | ▂▂▂▂▂▂▂▂▂▂▂▂▂▂▂▂▂▂▂▂▃▃▃▂▂ |
| ENDOCR PRACT | 2019 | 3.64 | **2019** | 2023 | ▂▂▂▂▂▂▂▂▂▂▂▂▂▂▂▂▂▂▂▂▃▃▃▃▃ |
| CELL PROLIFERAT | 2013 | 3.41 | **2019** | 2021 | ▂▂▂▂▂▂▂▂▂▂▂▂▂▂▂▂▂▂▂▂▃▃▃▂▂ |
| INT J MOL SCI | 2018 | 39.5 | **2020** | 2023 | ▂▂▂▂▂▂▂▂▂▂▂▂▂▂▂▂▂▂▂▂▂▃▃▃▃ |
| FRONT ENDOCRINOL | 2017 | 21.5 | **2020** | 2023 | ▂▂▂▂▂▂▂▂▂▂▂▂▂▂▂▂▂▂▂▂▂▃▃▃▃ |
| J CLIN MED | 2020 | 19.04 | **2020** | 2023 | ▂▂▂▂▂▂▂▂▂▂▂▂▂▂▂▂▂▂▂▂▂▃▃▃▃ |
| FRONT ONCOL | 2016 | 16.74 | **2020** | 2023 | ▂▂▂▂▂▂▂▂▂▂▂▂▂▂▂▂▂▂▂▂▂▃▃▃▃ |
| JAMA ONCOL | 2018 | 15.14 | **2020** | 2023 | ▂▂▂▂▂▂▂▂▂▂▂▂▂▂▂▂▂▂▂▂▂▃▃▃▃ |
| TRANSL ANDROL UROL | 2017 | 14.49 | **2020** | 2023 | ▂▂▂▂▂▂▂▂▂▂▂▂▂▂▂▂▂▂▂▂▂▃▃▃▃ |
| CELL REP | 2014 | 13.55 | **2020** | 2023 | ▂▂▂▂▂▂▂▂▂▂▂▂▂▂▂▂▂▂▂▂▂▃▃▃▃ |
| INT J SURG | 2015 | 13.27 | **2020** | 2023 | ▂▂▂▂▂▂▂▂▂▂▂▂▂▂▂▂▂▂▂▂▂▃▃▃▃ |
| PLOS ONE | 2010 | 11.3 | **2020** | 2023 | ▂▂▂▂▂▂▂▂▂▂▂▂▂▂▂▂▂▂▂▂▂▃▃▃▃ |
| CELLS-BASEL | 2020 | 10.82 | **2020** | 2023 | ▂▂▂▂▂▂▂▂▂▂▂▂▂▂▂▂▂▂▂▂▂▃▃▃▃ |
| ONCOL LETT | 2018 | 9.16 | **2020** | 2023 | ▂▂▂▂▂▂▂▂▂▂▂▂▂▂▂▂▂▂▂▂▂▃▃▃▃ |
| STEM CELL RES | 2020 | 8.48 | **2020** | 2023 | ▂▂▂▂▂▂▂▂▂▂▂▂▂▂▂▂▂▂▂▂▂▃▃▃▃ |
| DEVELOPMENT | 2009 | 8.25 | **2020** | 2023 | ▂▂▂▂▂▂▂▂▂▂▂▂▂▂▂▂▂▂▂▂▂▃▃▃▃ |
| STEM CELL REP | 2020 | 7.58 | **2020** | 2023 | ▂▂▂▂▂▂▂▂▂▂▂▂▂▂▂▂▂▂▂▂▂▃▃▃▃ |
| SYST BIOL REPROD MED | 2020 | 7.57 | **2020** | 2021 | ▂▂▂▂▂▂▂▂▂▂▂▂▂▂▂▂▂▂▂▂▂▃▃▂▂ |
| CELL METAB | 2016 | 7.51 | **2020** | 2023 | ▂▂▂▂▂▂▂▂▂▂▂▂▂▂▂▂▂▂▂▂▂▃▃▃▃ |
| J PEDIATR SURG | 2012 | 7.46 | **2020** | 2023 | ▂▂▂▂▂▂▂▂▂▂▂▂▂▂▂▂▂▂▂▂▂▃▃▃▃ |
| BIOMED PHARMACOTHER | 2000 | 7.35 | **2020** | 2023 | ▂▂▂▂▂▂▂▂▂▂▂▂▂▂▂▂▂▂▂▂▂▃▃▃▃ |
| CANCER MANAG RES | 2020 | 7.14 | **2020** | 2023 | ▂▂▂▂▂▂▂▂▂▂▂▂▂▂▂▂▂▂▂▂▂▃▃▃▃ |
| BMC GENOMICS | 2020 | 7.03 | **2020** | 2021 | ▂▂▂▂▂▂▂▂▂▂▂▂▂▂▂▂▂▂▂▂▂▃▃▂▂ |
| J OVARIAN RES | 2020 | 6.71 | **2020** | 2023 | ▂▂▂▂▂▂▂▂▂▂▂▂▂▂▂▂▂▂▂▂▂▃▃▃▃ |
| OXID MED CELL LONGEV | 2020 | 6.49 | **2020** | 2021 | ▂▂▂▂▂▂▂▂▂▂▂▂▂▂▂▂▂▂▂▂▂▃▃▂▂ |
| EMBO J | 2001 | 5.91 | **2020** | 2023 | ▂▂▂▂▂▂▂▂▂▂▂▂▂▂▂▂▂▂▂▂▂▃▃▃▃ |
| BBA-MOL BASIS DIS | 2020 | 5.8 | **2020** | 2023 | ▂▂▂▂▂▂▂▂▂▂▂▂▂▂▂▂▂▂▂▂▂▃▃▃▃ |
| GENE | 2000 | 5.57 | **2020** | 2021 | ▂▂▂▂▂▂▂▂▂▂▂▂▂▂▂▂▂▂▂▂▂▃▃▂▂ |
| CELL DEATH DIS | 2016 | 5.16 | **2020** | 2023 | ▂▂▂▂▂▂▂▂▂▂▂▂▂▂▂▂▂▂▂▂▂▃▃▃▃ |
| ACTA OBSTET GYN SCAN | 2005 | 4.87 | **2020** | 2023 | ▂▂▂▂▂▂▂▂▂▂▂▂▂▂▂▂▂▂▂▂▂▃▃▃▃ |
| DEV BIOL | 2008 | 4.78 | **2020** | 2021 | ▂▂▂▂▂▂▂▂▂▂▂▂▂▂▂▂▂▂▂▂▂▃▃▂▂ |
| CANCER SCI | 2005 | 4.47 | **2020** | 2023 | ▂▂▂▂▂▂▂▂▂▂▂▂▂▂▂▂▂▂▂▂▂▃▃▃▃ |
| CANCER DISCOV | 2020 | 4.1 | **2020** | 2023 | ▂▂▂▂▂▂▂▂▂▂▂▂▂▂▂▂▂▂▂▂▂▃▃▃▃ |
| ANTIOXID REDOX SIGN | 2020 | 4.1 | **2020** | 2023 | ▂▂▂▂▂▂▂▂▂▂▂▂▂▂▂▂▂▂▂▂▂▃▃▃▃ |
| AM J HEMATOL | 2004 | 4.03 | **2020** | 2021 | ▂▂▂▂▂▂▂▂▂▂▂▂▂▂▂▂▂▂▂▂▂▃▃▂▂ |
| ACTAS UROL ESP | 2020 | 4.01 | **2020** | 2023 | ▂▂▂▂▂▂▂▂▂▂▂▂▂▂▂▂▂▂▂▂▂▃▃▃▃ |
| REPRODUCTIVE MEDICINE AND BIOLOGY | 2020 | 3.73 | **2020** | 2023 | ▂▂▂▂▂▂▂▂▂▂▂▂▂▂▂▂▂▂▂▂▂▃▃▃▃ |
| HUM REPROD OPEN | 2021 | 20.17 | **2021** | 2023 | ▂▂▂▂▂▂▂▂▂▂▂▂▂▂▂▂▂▂▂▂▂▂▃▃▃ |
| CANCER MED-US | 2019 | 12.17 | **2021** | 2023 | ▂▂▂▂▂▂▂▂▂▂▂▂▂▂▂▂▂▂▂▂▂▂▃▃▃ |
| JAMA NETW OPEN | 2021 | 12.09 | **2021** | 2023 | ▂▂▂▂▂▂▂▂▂▂▂▂▂▂▂▂▂▂▂▂▂▂▃▃▃ |
| REPROD SCI | 2019 | 9.52 | **2021** | 2023 | ▂▂▂▂▂▂▂▂▂▂▂▂▂▂▂▂▂▂▂▂▂▂▃▃▃ |
| FRONT IMMUNOL | 2021 | 8.95 | **2021** | 2023 | ▂▂▂▂▂▂▂▂▂▂▂▂▂▂▂▂▂▂▂▂▂▂▃▃▃ |
| MOLECULES | 2021 | 8.95 | **2021** | 2023 | ▂▂▂▂▂▂▂▂▂▂▂▂▂▂▂▂▂▂▂▂▂▂▃▃▃ |
| STEM CELL RES THER | 2015 | 8.53 | **2021** | 2023 | ▂▂▂▂▂▂▂▂▂▂▂▂▂▂▂▂▂▂▂▂▂▂▃▃▃ |
| NAT METHODS | 2021 | 8.51 | **2021** | 2023 | ▂▂▂▂▂▂▂▂▂▂▂▂▂▂▂▂▂▂▂▂▂▂▃▃▃ |
| AGING-US | 2021 | 8.06 | **2021** | 2023 | ▂▂▂▂▂▂▂▂▂▂▂▂▂▂▂▂▂▂▂▂▂▂▃▃▃ |
| CANCER EPIDEMIOL | 2018 | 7.97 | **2021** | 2023 | ▂▂▂▂▂▂▂▂▂▂▂▂▂▂▂▂▂▂▂▂▂▂▃▃▃ |
| REPROD DOMEST ANIM | 2021 | 7.81 | **2021** | 2023 | ▂▂▂▂▂▂▂▂▂▂▂▂▂▂▂▂▂▂▂▂▂▂▃▃▃ |
| JOVE-J VIS EXP | 2021 | 7.29 | **2021** | 2023 | ▂▂▂▂▂▂▂▂▂▂▂▂▂▂▂▂▂▂▂▂▂▂▃▃▃ |
| SEMIN CELL DEV BIOL | 2017 | 6.95 | **2021** | 2023 | ▂▂▂▂▂▂▂▂▂▂▂▂▂▂▂▂▂▂▂▂▂▂▃▃▃ |
| BMC PUBLIC HEALTH | 2019 | 6.83 | **2021** | 2023 | ▂▂▂▂▂▂▂▂▂▂▂▂▂▂▂▂▂▂▂▂▂▂▃▃▃ |
| FRONT SURG | 2021 | 6.77 | **2021** | 2023 | ▂▂▂▂▂▂▂▂▂▂▂▂▂▂▂▂▂▂▂▂▂▂▃▃▃ |
| HEAD NECK PATHOL | 2021 | 6.25 | **2021** | 2023 | ▂▂▂▂▂▂▂▂▂▂▂▂▂▂▂▂▂▂▂▂▂▂▃▃▃ |
| EJSO-EUR J SURG ONC | 2010 | 6.12 | **2021** | 2023 | ▂▂▂▂▂▂▂▂▂▂▂▂▂▂▂▂▂▂▂▂▂▂▃▃▃ |
| BIOFABRICATION | 2021 | 5.73 | **2021** | 2023 | ▂▂▂▂▂▂▂▂▂▂▂▂▂▂▂▂▂▂▂▂▂▂▃▃▃ |
| J OBSTET GYNAECOL RE | 2021 | 5.73 | **2021** | 2023 | ▂▂▂▂▂▂▂▂▂▂▂▂▂▂▂▂▂▂▂▂▂▂▃▃▃ |
| WORLD J GASTROENTERO | 2006 | 5.66 | **2021** | 2023 | ▂▂▂▂▂▂▂▂▂▂▂▂▂▂▂▂▂▂▂▂▂▂▃▃▃ |
| NAT BIOTECHNOL | 2001 | 5.57 | **2021** | 2023 | ▂▂▂▂▂▂▂▂▂▂▂▂▂▂▂▂▂▂▂▂▂▂▃▃▃ |
| COCHRANE DB SYST REV | 2012 | 5.44 | **2021** | 2023 | ▂▂▂▂▂▂▂▂▂▂▂▂▂▂▂▂▂▂▂▂▂▂▃▃▃ |
| ASIAN PAC J CANCER P | 2013 | 5.23 | **2021** | 2023 | ▂▂▂▂▂▂▂▂▂▂▂▂▂▂▂▂▂▂▂▂▂▂▃▃▃ |
| J THORAC CARDIOV SUR | 2006 | 5.22 | **2021** | 2023 | ▂▂▂▂▂▂▂▂▂▂▂▂▂▂▂▂▂▂▂▂▂▂▃▃▃ |
| JAMA SURG | 2021 | 4.92 | **2021** | 2023 | ▂▂▂▂▂▂▂▂▂▂▂▂▂▂▂▂▂▂▂▂▂▂▃▃▃ |
| J EXP CLIN CANC RES | 2021 | 4.92 | **2021** | 2023 | ▂▂▂▂▂▂▂▂▂▂▂▂▂▂▂▂▂▂▂▂▂▂▃▃▃ |
| FRONT PHARMACOL | 2021 | 4.92 | **2021** | 2023 | ▂▂▂▂▂▂▂▂▂▂▂▂▂▂▂▂▂▂▂▂▂▂▃▃▃ |
| CUAJ-CAN UROL ASSOC | 2013 | 4.7 | **2021** | 2023 | ▂▂▂▂▂▂▂▂▂▂▂▂▂▂▂▂▂▂▂▂▂▂▃▃▃ |
| J GASTROEN HEPATOL | 1999 | 4.35 | **2021** | 2023 | ▂▂▂▂▂▂▂▂▂▂▂▂▂▂▂▂▂▂▂▂▂▂▃▃▃ |
| CELL MOL LIFE SCI | 2009 | 4.17 | **2021** | 2023 | ▂▂▂▂▂▂▂▂▂▂▂▂▂▂▂▂▂▂▂▂▂▂▃▃▃ |
| INT J GYNECOL CANCER | 2017 | 3.94 | **2021** | 2023 | ▂▂▂▂▂▂▂▂▂▂▂▂▂▂▂▂▂▂▂▂▂▂▃▃▃ |
| NUCLEIC ACIDS RES | 2007 | 3.9 | **2021** | 2023 | ▂▂▂▂▂▂▂▂▂▂▂▂▂▂▂▂▂▂▂▂▂▂▃▃▃ |
| J HEPATOL | 2006 | 3.85 | **2021** | 2023 | ▂▂▂▂▂▂▂▂▂▂▂▂▂▂▂▂▂▂▂▂▂▂▃▃▃ |
